# The jet-like chromatin structure defines active secondary metabolism in fungi

**DOI:** 10.1093/nar/gkae131

**Published:** 2024-02-26

**Authors:** Wenyong Shao, Jingrui Wang, Yueqi Zhang, Chaofan Zhang, Jie Chen, Yun Chen, Zhangjun Fei, Zhonghua Ma, Xuepeng Sun, Chen Jiao

**Affiliations:** State Key Laboratory of Rice Biology, Key Laboratory of Molecular Biology of Crop Pathogens and Insects, Institute of Biotechnology, Zhejiang University, Hangzhou 310058, Zhejiang, China; State Key Laboratory of Rice Biology, Key Laboratory of Molecular Biology of Crop Pathogens and Insects, Institute of Biotechnology, Zhejiang University, Hangzhou 310058, Zhejiang, China; State Key Laboratory of Rice Biology, Key Laboratory of Molecular Biology of Crop Pathogens and Insects, Institute of Biotechnology, Zhejiang University, Hangzhou 310058, Zhejiang, China; State Key Laboratory of Rice Biology, Key Laboratory of Molecular Biology of Crop Pathogens and Insects, Institute of Biotechnology, Zhejiang University, Hangzhou 310058, Zhejiang, China; Collaborative Innovation Center for Efficient and Green Production of Agriculture in Mountainous Areas of Zhejiang Province, College of Horticulture Science, Zhejiang A&F University, Hangzhou 311300, Zhejiang, China; National Joint Engineering Laboratory of Biopesticide Preparation, College of Forestry and Biotechnology, Zhejiang A&F University, Hangzhou 311300, Zhejiang, China; State Key Laboratory of Rice Biology, Key Laboratory of Molecular Biology of Crop Pathogens and Insects, Institute of Biotechnology, Zhejiang University, Hangzhou 310058, Zhejiang, China; Boyce Thompson Institute, Cornell University, Ithaca, NY 14853, USA; State Key Laboratory of Rice Biology, Key Laboratory of Molecular Biology of Crop Pathogens and Insects, Institute of Biotechnology, Zhejiang University, Hangzhou 310058, Zhejiang, China; Collaborative Innovation Center for Efficient and Green Production of Agriculture in Mountainous Areas of Zhejiang Province, College of Horticulture Science, Zhejiang A&F University, Hangzhou 311300, Zhejiang, China; State Key Laboratory of Rice Biology, Key Laboratory of Molecular Biology of Crop Pathogens and Insects, Institute of Biotechnology, Zhejiang University, Hangzhou 310058, Zhejiang, China

## Abstract

Eukaryotic genomes are spatially organized within the nucleus in a nonrandom manner. However, fungal genome arrangement and its function in development and adaptation remain largely unexplored. Here, we show that the high-order chromosome structure of *Fusarium graminearum* is sculpted by both H3K27me3 modification and ancient genome rearrangements. Active secondary metabolic gene clusters form a structure resembling chromatin jets. We demonstrate that these jet-like domains, which can propagate symmetrically for 54 kb, are prevalent in the genome and correlate with active gene transcription and histone acetylation. Deletion of *GCN5*, which encodes a core and functionally conserved histone acetyltransferase, blocks the formation of the domains. Insertion of an exogenous gene within the jet-like domain significantly augments its transcription. These findings uncover an interesting link between alterations in chromatin structure and the activation of fungal secondary metabolism, which could be a general mechanism for fungi to rapidly respond to environmental cues, and highlight the utility of leveraging three-dimensional genome organization in improving gene transcription in eukaryotes.

## Graphical Abstract

**Graphical Abstract:**
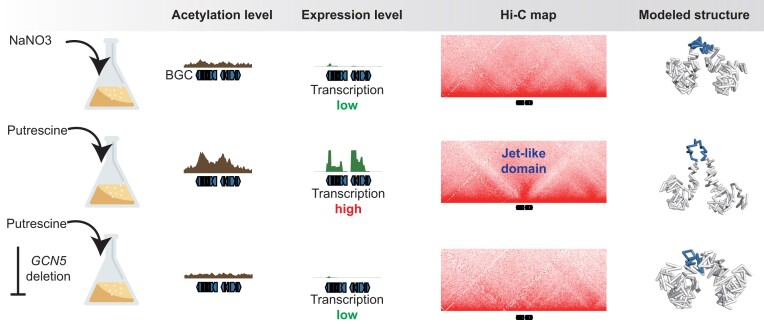

## Introduction

The eukaryotic genome is packaged hierarchically into multiscale structural units (1). Chromosomes often occupy distinct subnuclear territories (2), with transcriptionally active regions located at their surface (3). Compartments, topologically associating domains (TADs) and loops are typic chromatin architectures commonly found in plants and animals (1,4,5). TADs define the boundaries of regulatory domains (6), within which promoters interact with enhancers to form loops (7) or stripes when enhancers are superactive (8). Compared with TADs, which are relatively conserved across tissues and species (9,10), chromatin structures are more extensively reorganized locally (11). The fungal genomes often show a Rabl-like chromosome configuration, in which centromeres cluster and chromosome arms are organized in parallel (12–16). Self-associating domains in fungi are notably shorter, with sizes scaled by gene number rather than by genomic distance (17). Unlike in animals and plants, chromatin structures and their functions in fungi remain largely unexplored.

Fungi are known to produce a vast array of secondary metabolites (SMs) that are critical for their survival and adaptation (18). Biosynthesis of SMs is regulated by a complex interplay of environmental cues such as temperature, light, humidity, pH and nutrient availability (19–21). Genes involved in secondary metabolism are rapidly induced or repressed in response to these environmental signals, leading to significant changes in SM production within a short time (22). The rapid response is likely facilitated by the presence of clustered biosynthetic genes, which are often located in proximity to regulatory elements (23). However, transcriptional co-regulation of genes does not necessarily require physical proximity on chromosomes (24), indicating that the evolution of biosynthetic gene clusters (BGCs) may provide additional advantages. It has been proposed that gene clustering can establish local chromatin environments that are more permissive for transcriptional activation or repression, thus enhancing the efficiency of gene expression controlled by transcriptional and epigenetic machinery (25,26). Epigenetic mechanisms, such as histone modifications, allow the fine-tuning and coordination of temporal gene expression in fungi (27,28). Among them, the trimethylation of histone H3 lysine 27 (H3K27me3), a conserved epigenetic mark of facultative heterochromatin and gene silencing, has been found to be enriched in BGCs in several fungal species (29–32). Deletion of histone methyltransferases responsible for H3K27me3 leads to the upregulation of genes involved in secondary metabolism and an increase in SM production (29,30). Interestingly, epigenetic modifications, including H3K27me3, acetylation of histone H3 lysine 27 (H3K27ac) and trimethylation of histone H3 lysine 9, have been shown to play a role in genome compartmentalization in mammals (33), and contribute to a ‘layer cake’ model of chromosome 3D organization in plants and metazoans (34). However, the impact of epigenetic modifications on the spatial organization of fungal genomes and secondary metabolism remains unclear.

To understand whether and how fungal chromosome architectures affect SM production, we focused on *Fusarium graminearum*, a devastating fungal pathogen causing *Fusarium* head blight in wheat. *Fusarium* *graminearum* produces a number of SMs, among which the deoxynivalenol (DON) toxin poses a great threat to public health (35). Substantial studies have unveiled key biosynthetic genes (*Tri* genes) and various regulators involved in DON production (20). In this study, we report for the first time that the active DON BGC forms a jet-like domain, which likely facilitates *Tri* gene transcription. Moreover, the jet-like domains are prevalent in the genome and correlate with active gene transcription and histone acetylation. Deletion of *GCN5*, which encodes a core and functionally conserved histone acetyltransferase (HAT), results in the absence of the domains. Notably, insertion of an exogenous gene within the jet-like domain significantly enhances its transcription. Collectively, our results establish a conceptual framework for understanding the regulation of chromatin structures and secondary metabolism, and highlight the utility of genome organization in improving target gene transcription in fungi. In addition, we elucidated the regulatory role of a previously uncharacterized acetyltransferase in DON biosynthesis, providing critical information for future management of this fungal pathogen.

## Materials and methods

### Strain and medium

The *F*. *graminearum* strain PH-1 was used in this study. The PH-1 was cultured in PDA (200 g potato, 20 g glucose and 18 g agar per 1 l ddH_2_O) or PDB (200 g potato and 20 g glucose per 1 l ddH_2_O) medium for morphological examination. The putrescine medium (30 g sucrose, 1 g KH_2_PO_4_, MgSO_4_·7H_2_O, 0.5 g KCl, 0.01 g FeSO_4_·7H_2_O and 1.5 g putrescine per 1 l ddH_2_O) and NaNO_3_ medium (30 g sucrose, 1 g KH_2_PO_4_, MgSO_4_**·**7H_2_O, 0.5 g KCl, 0.01 g FeSO_4_**·**7H_2_O and 2 g NaNO_3_ per 1 l ddH_2_O) were used to culture the fungus for DON measurement and sequencing library preparation.

### qRT-PCR assay

Ten mycelial plugs, taken from the margin of PH-1 colony grown on PDA for 3 days at 25°C, were added into a 2-ml centrifuge tube containing one steel ball and 1 ml PDB. The mixture was ground using a tissue lyser and then transferred to a 250-ml triangular flask containing 200 ml of putrescine or NaNO_3_ medium. The flask was incubated in a shaker at 28°C and 180 rpm for 18, 24, 30, 32, 34, 36, 38 and 40 h. After incubation, the mycelia were collected and ground into powder in liquid nitrogen. Total RNA was isolated using the RNA isolation kit (Tiangen, China). Approximately 0.8 μg of total RNA from each sample was used for complementary DNA (cDNA) synthesis with the HiScript II 1st strand cDNA synthesis kit (Vazyme, China). Gene expression was quantified using HiScipt II Q RT SuperMix for qPCR (+gDNA wiper) kit (Vazyme) with the primer sequences listed in Supplementary Table S1. The actin gene was used as the reference. Each experiment was repeated three times.

### DON production assay

PH-1 was cultured under the condition mentioned in the quantitative reverse transcription polymerase chain reaction (qRT-PCR) assay section. For each sample, the mycelia and nutrient solution were collected using gauze filtration. In the time-series experiments, 50 μl of the nutrient solution was taken and used to measure the concentration of DON using the DON assay kit (Weisai, China). To measure DON concentration more accurately in the gene deletion mutant, 1 ml of cell-free supernatant was subjected to filtration and subsequently purified by passing through SampliQ Amino (NH_2_) solid-phase extraction columns (Agilent Technologies). The purified extract (4 ml) was evaporated to dryness under a nitrogen stream. The resulting residue was dissolved in 1 ml mixture of methanol and water (40:60, v/v) and subjected to centrifugation at 10 000 rpm. The final solution was then analyzed by using liquid chromatography–tandem mass spectrometry, and the concentration of DON was estimated based on the curve of standard DON solutions (Sigma–Aldrich, USA). The DON production *in vitro* was expressed as a ratio of DON concentration weight to mycelia dry weight (μg/g) (36). The entire experiment was repeated three times.

### Transcriptome sequencing and analysis

Total RNA was extracted using the RNA isolation kit (Tiangen, China). The quantity and integrity of the RNA samples were assessed using the RNA Nano 6000 Assay Kit on a Bioanalyzer 2100 system (Agilent Technologies, Santa Clara, CA, USA). RNA sequencing (RNA-seq) library was prepared from the high-quality RNA samples (RNA integrity number ≥8) with the NEBNext Ultra II RNA Library Prep Kit for Illumina (NEB) following the manufacturer’s instructions. Subsequently, the quality of the libraries was assessed using Qubit 2.0 and Bioanalyzer 2100, and the libraries were sequenced on an Illumina NovaSeq platform under the 2 × 150-bp mode.

The raw RNA-seq reads were processed to remove adapters and low-quality bases with Trimmomatic (v0.39) (37). The cleaned reads were mapped to the PH-1 reference genome (FungiDB release 58, https://fungidb.org/fungidb/app) using HISAT2 (v.2.2.1) (38). Finally, the unique alignments were counted using HTSeq-count (v.0.11.3) (39) and the differentially expressed genes were identified with DESeq2 (v.1.36.0) (40) under the cutoff of adjusted *P*-value ≤0.05 and |log_2_(fold change)| ≥ 1. Coverage tracks of gene expression of 1-kb genomic bin were calculated using bamCoverage from the deepTools package (v3.5.0) (41) with the read coverage normalized in FPKM (fragments per kilobase of transcript per million mapped fragments).

### Nuclei isolation

A total of 0.25 g of mycelia were ground into powder using a mortar and pestle in liquid nitrogen. The powder was then incubated with 4 ml lysis buffer (20 mM Tris–HCl, pH 7.5, 20 mM KCl, 2 mM EDTA, 2.5 mM MgCl_2_, 25% glycerol and 250 mM sucrose) for 10 min on ice to release the nuclei. The mixture was subsequently filtered through Miracloth to remove any cell debris, and the filtrate was centrifuged at 1500 × *g* and 4°C for 10 min. The resulting supernatant was carefully transferred to a new 1.5-ml tube and centrifuged again at 4°C and 13 000 rpm for 15 min. The sediment containing the cell nuclei was then resuspended in 4 ml of the NRBT buffer (20 mM Tris–HCl, pH 7.5, 2.5 mM MgCl_2_, 25% glycerol and 0.2% Triton X-100). The suspension was centrifuged at 1500 × *g* and 4°C for 10 min. This step was repeated four times to ensure thorough washing of the nuclei. Consequently, the NRBT buffer was removed, and the sediment was resuspended in lysis buffer for downstream experiments.

### ATAC-seq library preparation and sequencing

Intact nuclei were isolated from 0.25 g of mycelia in each experiment. The nuclei were pelleted by centrifugation and resuspended in 20 μl of 1× TTBL buffer (VAHTS, TD501). The integrity and quantity of the isolated nuclei were carefully assessed prior to further experiments. The ATAC-seq libraries were prepared using the TruePrep DNA Library Prep Kit V2 for Illumina (Vazyme, China). In each run, ∼10 000 nuclei were subjected to Tn5 transposase treatment. The transposition reaction was performed at 37°C for 30 min. After that, DNA fragments were conjugated with adapters, followed by PCR amplification for 10–13 cycles. The DNA fragments were then size selected with VAHTS DNA Clean Beads (Vazyme, China), and the final libraries were quality assessed with Bioanalyzer 2100 and sequenced on an Illumina NovaSeq platform under the 2 × 150-bp mode.

### CUT&Tag library preparation and sequencing

CUT&Tag libraries were constructed using the Hyperactive Universal CUT&Tag Assay Kit for Illumina (TD903, Vazyme, China). Briefly, the intact nuclei were isolated from 0.25 g of mycelia of each treatment and resuspended in 10 μl binding buffer with ConA beads. After incubating for 10 min at 25°C, the ConA bead–nucleus complex was pelleted by hopper magnet and resuspended in 50 μl of cold antibody buffer. Then, 2.5 μl of primary antibody of H3K4me1, H3K4me3, H3K9ac, H3K27ac (A2355, A2357, A7255 and A7253, Abclonal) and H3K27me3 (ab195477, Abcam) was added, respectively, and the reaction solution was incubated at 4°C overnight. The ConA bead–nucleus complex was pelleted again by hopper magnet and resuspended in 50 μl Dig-wash buffer containing secondary antibody (1:100 dilution, Vazyme). After incubation at 25°C for 60 min, the ConA bead–nucleus complex was pelleted and washed with 200 μl of Dig-wash buffer three times. Subsequently, the complex was resuspended in 100 μl of Dig-300 buffer containing 2 μl of pA/G-Tn5 and kept for reaction at 25°C for 60 min. After that, the beads were pelleted and washed with 200 μl of Dig-300 buffer three times. The beads were resuspended in 50 μl of Dig-300 buffer containing 10 μl of 5× TTBL and incubated at 37°C for 60 min. Then, 5 μl of proteinase K, 100 μl of buffer L/B and 20 μl of DNA extract beads were added and incubated at 55°C for 10 min. The beads were pelleted and washed with 200 μl of WA buffer once and WB buffer twice. After the beads were air dried, the DNA fragments binding on beads were eluted with 22 μl of pure water, 20 μl of which was used for library preparation. The libraries were sequenced on an Illumina NovaSeq platform under the 2 × 150-bp mode.

### Data analysis of ATAC-seq and CUT&Tag sequencing

Raw reads of ATAC-seq and CUT&Tag sequencing were processed to remove adapters using Cutadapt (v.4.1) (42). The cleaned reads (≥20 bp) were aligned to the PH-1 genome using Bowtie2 (v.2.4.5) (43), and the unique alignments of ATAC-seq data were positionally shifted using alignmentSieve from the deepTools package with the parameter ‘--ATACshift’. Peaks were called using MACS2 (v2.2.7) (44) with ‘--keep-dup all’ parameter for ATAC-seq, H3K4me3, H3K9ac and H3K27ac, and ‘--keep-dup all --broad’ parameter for H3K4me1 and H3K27me3. Coverage tracks with a bin size of 50 bp were calculated using the bamCoverage and FPKM normalized data. The generated datasets were visualized by pyGenomeTracks (45).

### Hi-C library preparation and data analysis

The mycelia of PH-1 were fixed in a 1% (v/v) formaldehyde solution for 30 min at room temperature. The cross-linking reaction was terminated by the addition of a glycine solution. The fixed mycelia were resuspended in 1 ml of lysis buffer and incubated on ice for 20 min. Nuclei were pelleted through centrifugation at 600 × *g* for 5 min at 4°C, followed by thorough washing with 1 ml of lysis buffer (46). Once pelleted, the nuclei were resuspended in 400 μl of a restriction enzyme buffer and transferred to a safe lock tube. The chromatin was solubilized by the addition of dilute sodium dodecyl sulfate (SDS) and incubated at 65°C for 10 min. After quenching the SDS with Triton X-100, overnight digestion was carried out using the restriction endonuclease MboI at 37°C on a rocking platform. The resulting cohesive ends were filled with a biotin marker to generate blunt ends, followed by ligation using T4 DNA ligase (New England Biolabs Inc.). DNA cross-linking was then disrupted using proteinase K (Thermo Fisher, Waltham, MA), and DNA was purified through phenol–chloroform extraction. Nonligated fragment ends carrying biotin labels were removed using T4 DNA polymerase. Subsequently, the DNA fragments were sheared by sonication to achieve a size range of 200–600 bp. The fragments were end-repaired by a mixture of T4 DNA polymerase, T4 polynucleotide kinase and Klenow DNA polymerase. Biotin-labeled DNA fragments were purified through pulldown using streptavidin magnetic beads. The DNA fragment ends were then subjected to A-tailing using Klenow DNA polymerase, followed by the addition of Illumina paired-end sequencing adapters using a ligation mix (47). Finally, the Hi-C libraries were amplified by PCR with 12–14 cycles and sequenced on an Illumina NovaSeq platform under the 2 × 150-bp mode.

Raw Hi-C reads were processed to remove adapters and low-quality sequences. The cleaned Hi-C reads were aligned to the PH-1 genome using bwa (v.0.7.17) (48) with the parameters set to ‘-A1 -B4 -E50 -L0’. The paired alignments were then processed using HiCExplorer (v.3.7.2) (49) to generate the contact matrices at the resolution of 1 kb. Hi-C matrices with different bin resolutions were generated using the HicMergeMatrixBins tool in HiCExplorer. All interaction matrices were normalized using HicNormalize, followed by Knight–Ruiz matrix balancing using hicCorrectMatrix. Principal component analysis (PCA) was performed on matrices with a bin size of 25 kb using hicPCA with the Lieberman method. Average Hi-C contacts around selected genes or peaks were computed using HicAverageRegions. Hi-C data from biological replicates were combined before downstream analysis.

### Identification and analysis of the jet-like domain

The Hi-C interaction matrix was transformed into a dense format using the sparseToDense.py script from HiC-Pro (50). This matrix was used to calculate two metrics, namely the insulation score and the delta value (51), using the script matrix2insulation.pl (https://github.com/dekkerlab/cworld-dekker) with parameters set to ‘-is 10000 -ids 6000’. Specifically, to calculate the insulation score of each 1-kb bin, we slid a 10 kb × 10 kb (10 × 10 bins) square along the matrix diagonal and aggregated all signals within the square. The mean signal within the square was then assigned to the 1-kb diagonal bin and this procedure was then repeated for all 1-kb diagonal bins (Supplementary Figure S1A). The insulation score was then normalized relative to all of the insulation scores across each chromosome by calculating the log_2_ ratio of each bin’s insulation score and the mean of all insulation scores. Meanwhile, the delta value, which was defined as the difference between the mean insulation scores 3 kb to the left of the central bin and 3 kb to the right of the central bin, was calculated for each 1-kb diagonal bin (Supplementary Figure S1A). Bins at the peak of insulation score profile with a delta value equal to zero were extracted. For these bins, we further calculated the strength of insulation by measuring the difference of delta values between the local maximum (Δ_max_) and local minimum (Δ_min_) of the bin and removed those with the strength value <0.3. The preserved bins represent the center of candidate jet-like domains. Typical jet-like domains contain two stripes at ∼45° (left stripe) and ∼135° (right stripe) angles off the diagonal, which represent low chromatin interactions between the focal area and the center of the domain (Supplementary Figure S1B). To verify the presence of the stripes for each candidate domain, we aggregated the signals in the sliding squares based on the observed/expected matrix. For the left stripe, we performed two rounds of sliding, with the first starting at the center of the domain (higher interaction region) and the second starting immediately upstream of the domain center (lower interaction region). For each round, interaction signals from 10 squares (step size = 1 kb) were calculated. We used *t*-test to compare the interaction intensities between two sets of sliding squares, and a *P*-value <0.05 was used to define the presence of the stripe in the candidate domain. Similarly, the presence of the right stripe for each domain was verified by comparing sliding squares starting at or immediately downstream of the domain center. Consequently, domains with both stripes were preserved and plotted for a final manual examination (Supplementary Figure S1B).

To measure the length of the jet-like domain, we used the observed/expected matrix to aggregate signals within a 10 kb × 10 kb (10 × 10 bins) square, which moves perpendicularly away from the matrix diagonal starting at the center of each domain (52). The sliding square quantifies the genomic contacts between two flanking regions at varying distances. We applied the same technique to randomly selected bins to serve as a control. It has been observed that the neighboring regions of a jet-like domain center generally have more contacts compared to the control. The length of the domain is determined by the point at which the contact intensity of the two flanking regions from the domain area decreases to the level of the contacts in the control regions.

### Creation of gene deletion mutants

For gene deletion, the flanking sequences of the target gene were amplified from the genomic DNA with primers listed in Supplementary Table S1. The two amplified fragments were fused together with a hygromycin resistance gene cassette (HPH) through a double-joint PCR reaction (53). The construct was transferred to the protoplast of PH-1 following a modified protocol (54). In brief, 1 g of mycelia were suspended in 20 ml of 0.7 M NaCl buffer containing 0.1 g driselase, 0.2 g lysing enzymes and 0.2 g snailase (Sigma, St Louis, MO, USA) and incubated for 2 h at 30°C and 85 rpm. Protoplasts were separated from cell debris with filtration through four layers of lens tissue and washed twice with NaCl buffer. Then, the protoplasts were suspended sequentially in 10 ml of STC buffer and SPTC buffer and incubated with the gene deletion construct on ice for 30 min. Transformed protoplasts were added into 20 ml of the RM medium (1 g yeast extract, 1 g casein hydrolysate and 274 g sucrose per 1 l ddH_2_O). After incubation for 12 h at 25°C and 100 rpm, all culture was mixed with 200 ml of the melt PDA medium containing 100 μg/ml hygromycin B and poured into Petri plates for incubation at 25°C under dark condition. Antibiotic-resistant colonies were transferred to the PDA plate containing hygromycin B (100 μg/ml) and used in subsequent experiments.

### Virulence assay

The fungal strain was cultured in the MBL medium (a broth of 30 g mung beans boiled in 1 l of sterile water) at 25°C and 180 rpm for 5 days to induce the conidia (55). For inoculation, a 10 μl suspension of conidia (10^5^ conidia/ml) was injected into a floret located in the central section of the spikelet in single flowering wheat heads of the susceptible cultivar ‘Jimai22’. In contrast, for the control the heads were inoculated with 10 μl of sterile water. The experiment consisted of 20 replicates for each strain. Following inoculation, the plants were maintained under conditions of 100% humidity at a temperature of 22 ± 2°C for 2 days, after which they were transferred to a glasshouse environment (56). Fifteen days after inoculation, the number of infected spikelets in each inoculated wheat head was recorded. The experiment was repeated twice.

### Western blotting

Approximately 0.2 g of finely ground mycelia were resuspended in 1.5 ml of the isolation buffer (50 mM Tris–HCl, pH 7.5, 100 mM NaCl, 5 mM EDTA, 1% Triton X-100, 1% protease inhibitor cocktail). After homogenization with a vortex shaker, the lysate was centrifuged at 10 000 × *g* for 20 min at 4°C. The resulting proteins were separated on a 10% SDS–PAGE (polyacrylamide gel electrophoresis) gel and transferred to polyvinylidene fluoride (PVDF) membrane (Millipore, Billerica, MA, USA) with a Bio-Rad electroblotting apparatus. The PVDF membrane was incubated with the TBS-T buffer (8 g NaCl, 3 g Tris, 0.2 g KCl and 1 ml Tween 20 per 1 l pure water) containing 5% non-fat milk for 1 h at 25°C, and the PVDF membrane was then washed with the TBS-T buffer three times. Next, the membrane was incubated with the primary antibody of H3K9ac, H3K18ac, H3K27ac and H3 for 1 h at 25°C, respectively. After the PVDF membrane was washed with the TBS-T buffer three times, it was incubated with secondary antibodies for 1 h at 25°C. The PVDF membrane was washed again, and the chemical signal was detected using the western blotting detection system (GE, USA). The histone H3 protein was used as the reference (57). The antibodies of H3 (M1306-4, Huaan Biotechnology), H3K9ac, H3K18ac and H3K27ac (A7255, A7257 and A7253, Abclonal) were used at a 1:500 to 1:10 000 dilution for immunoblot analyses. The experiment was repeated twice.

### Other bioinformatics analyses

Gene Ontology term enrichment analysis was performed using GOATOOLS (v2.0) (58). Enrichment analysis of epigenetic modifications in A/B compartment was conducted with ChromHMM (v.1.23) (59). BGCs were predicted with antiSMASH (v.6.1.1) (60) with default parameters. CHESS (v.0.3.7) (61) was used to compare the two chromatin contact matrices at the 5 kb resolution using a window span of 100 kb and a step size of 20 kb. The 3D folding of chromosomes was reconstructed using LorDG (v.1.0) (62) with parameters ‘CONVERT_FACTOR = 0.6; LEARNING_RATE = 1.0; MAX_ITERATION = 2000’. The nucleotide diversity and recombination rate of *F*. *graminearum* were calculated based on the previously published datasets (63,64).

## Results

### Chromosome architecture of *F. graminearum* is shaped by epigenetic status and ancient genome rearrangements

To investigate the 3D organization of chromosomes during DON production, we cultured *F*. *graminearum* strain PH-1 in a toxin-inducing liquid medium supplemented with putrescine, a plant defense compound capable of stimulating DON production (65). The same medium with putrescine replaced by NaNO_3_ was used as a control. We set out to identify the optimal time point for sampling by monitoring biosynthetic gene expression as well as DON production in a time-series experiment (Supplementary Figure S2). DON production remained at a low level in the NaNO_3_ medium but elevated significantly from 34 h after incubation in the putrescine medium. Expression of DON biosynthetic genes, including *Tri1*, *Tri4*, *Tri5*, *Tri6* and *Tri10*, reached the peak at 36 h, at which DON content was increased by 7.5-fold (Supplementary Figure S2). Thus, mycelia at 36 h of cultivation were collected to profile transcriptomes, Hi-C-based chromosome interactions, chromatin accessibility (ATAC-seq) and histone modifications (i.e. H3K4me1, H3K4me3, H3K27me3, H3K9ac and H3K27ac). All the libraries were successfully constructed and yielded abundant high-quality and highly reproducible data for the 3D multi-omics analyses (Supplementary Table S2).

**Figure 1.**
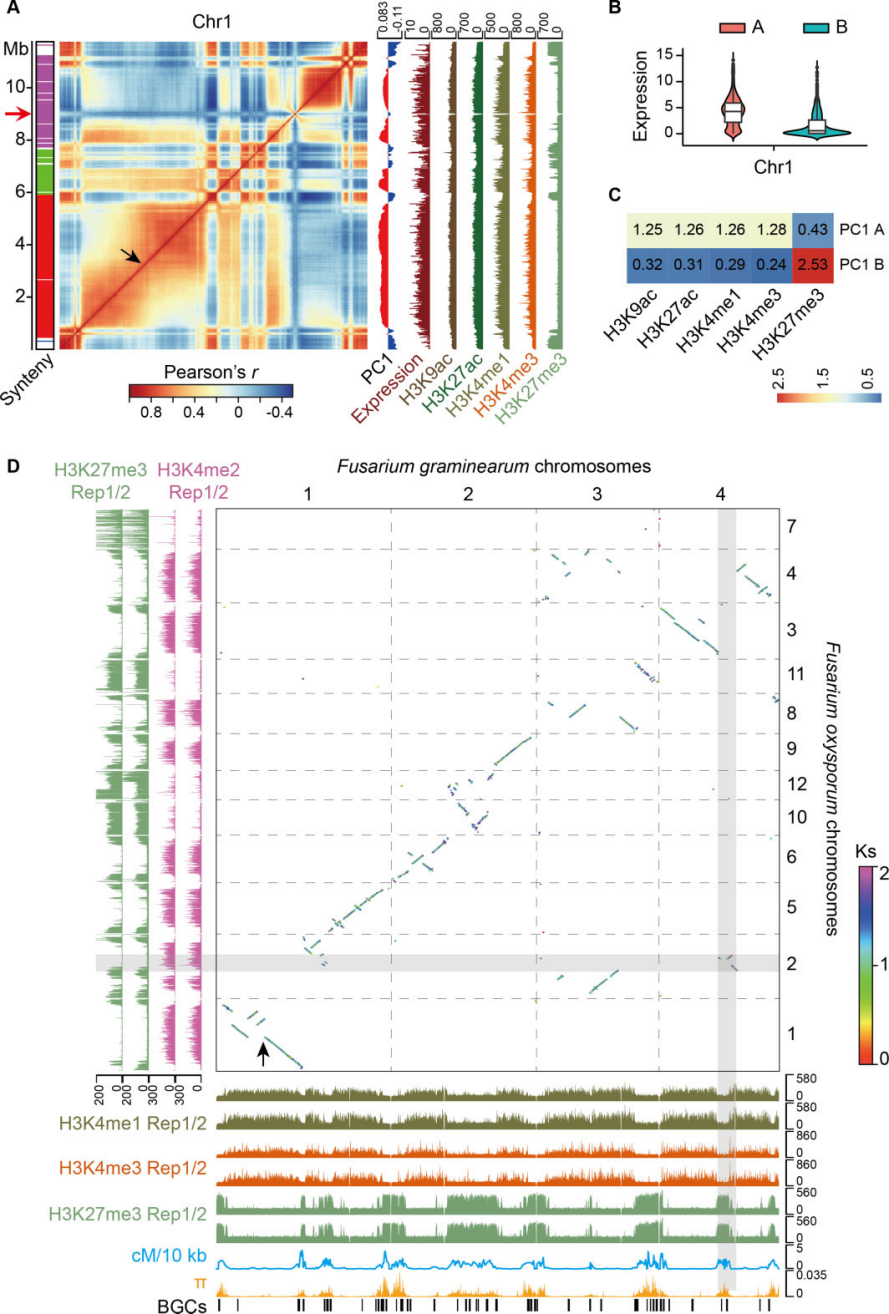
Epigenetic modifications and three-dimensional structure of the *F*. *graminearum* genome. (**A**) Epigenetic modifications and compartmentalization of chromosome 1 (Chr1). The heatmap shows Pearson correlation coefficient (*r*) of Hi-C interactions on Chr1 at 25 kb resolution. Colored bar on the left represents the genome synteny between *F*. *graminearum* and *F. oxysporum*, and each color corresponds to a single chromosome of *F. oxysporum* shown in Supplementary Figure S5, while the blank region indicates no synteny. The left arrow points to the centromere position. PCA-based compartmentalization as well as the status of gene expression and epigenetic modifications of each 25-kb window is displayed on the right. Biological replicates were combined to generate the plot. (**B**) Distribution of gene expression in A/B compartments defined by the first principal component (PC1). Expression of each gene was calculated as the averaged log_2_(FPKM + 1) of three biological replicates. (**C**) Enrichment analysis of epigenetic modifications in A/B compartments. Values indicate the fold of overrepresentation or underrepresentation of each mark in corresponding compartments. (**D**) Synteny and histone modifications of the two *Fusarium* genomes. Dot plot shows the synteny of the two genomes, with the syntenic fragments colored by their average *K*_s_ values. The recombination rate of *F*. *graminearum* genome was calculated as the genetic distance (cM) within the 10-kb window. The nucleotide diversity (*π*) was calculated based on a window size of 10 kb. Black arrows in panels (A) and (D) indicate genomic region where recombination has occurred.

The high-order structure of *F*. *graminearum* genome showed a Rabl-like configuration (Supplementary Figure S3), consistent with previous observation in other fungi (15). Genome compartments were inferred based on the PCA using Hi-C contact metrics, which showed that genomic regions occupied by Polycomb proteins, as manifested by the enrichment of H3K27me3 modifications, were frequently interacted (Figure 1A), leading to a compartmentalized genome with distinctive transcriptional activities (Figure 1B and C, and Supplementary Figure S4). The *F*. *graminearum* genome has evolved from an ancestral karyotype consisting of 11 or 12 chromosomes (66). We asked whether ancestral genome fissions and fusions had also influenced chromosome 3D organization in *F*. *graminearum*. To address this, we performed comparative genomic and epigenomic analyses with the early divergent relative *F*. *oxysporum* (67,68). Our data revealed that genomic regions exhibiting a high degree of collinearity between the two fungi were enriched with active histone modification H3K4me1/2 (Figure 1D). Conversely, the repressive H3K27me3 marked less conserved genomic regions and subtelomeres. These regions, which were enriched with BGCs, displayed frequent recombination and showed a high level of nucleotide diversity in the population (Figure 1D). Furthermore, we found that H3K27me3 was enriched at the breakpoints of genome rearrangements in *F*. *graminearum*. Notably, certain translocations, such as those on chromosome 4, were labeled with H3K27me3 despite being marked with active H3K4me2 in *F. oxysporum* (Figure 1D). In addition, we observed a breakdown of chromosome interactions at the regions where genomic synteny was disrupted by rearrangements (Figure 1A and D), and the intensity of chromosome interactions was much stronger in the continuous syntenic regions (e.g. chromosome 4) than in the frequently rearranged regions (e.g. chromosome 3; Supplementary Figures S4 and S5). These results indicate that genome rearrangements likely have affected both epigenetic modifications and genome 3D organization during the evolution of *F*. *graminearum* and other *Fusarium* species.

### DON production is associated with local chromatin reorganization

**Figure 2.**
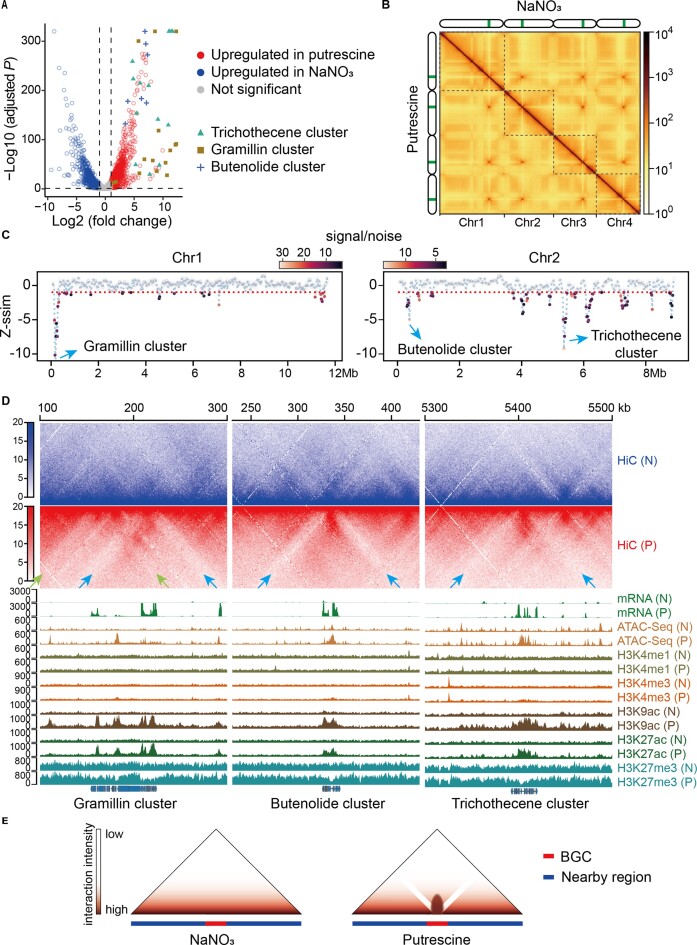
Genetic and epigenetic regulation of BGCs under DON-stimulating condition. (**A**) Differential gene expression of *F*. *graminearum* under the two culture conditions. (**B**) Physical interactions of *F*. *graminearum* genome revealed by Hi-C sequencing. Colored bars indicate the positions of centromeres. The resolution for the Hi-C map is 10 kb. (**C**) Structure changes of Chr1 and Chr2 under the two culture conditions. Similarity (*z*-normalized similarity score, *Z*-ssim) of Hi-C data generated from the two samples was assessed by CHESS using a sliding window of 100 kb. Highly dissimilar regions (*Z*-ssim ≤ −1) are indicated with the horizontal dash line. (**D**) Hi-C interaction maps of selected BGCs in putrescine (P) or NaNO_3_ (N) medium. Arrows of the same color indicate the jet-like domain overlapping the BGCs. Different colors of arrows indicate the presence of more than one jet-like domain. The status of gene expression, chromatin accessibility and histone modifications was calculated from all biological replicates. The resolution for the Hi-C map is 1 kb. (**E**) Graphic illustration showing alternation of local chromatin structure of BGC and its flanking regions (150–200 kb) under the two culture conditions. The panel shows the enhanced interaction within BGC (the middle bar) and reduced interaction between BGC and its flanking regions in the putrescine condition.

Transcriptome analysis of *F*. *graminearum* cultured in the putrescine and NaNO_3_ media revealed 1349 and 2873 genes that were preferentially expressed in the two conditions, respectively (Supplementary Table S3). Upregulated genes in the putrescine medium were significantly enriched with those involved in the secondary metabolism (Supplementary Figure S6), and genes in the biosynthetic clusters of DON, gramillin and butenolide displayed particularly significant expression differences (Figure 2A). Comparison of chromatin accessibility identified by ATAC-seq showed that promoter regions of BGCs exhibited much higher levels of open accessibility in putrescine treatment, but such differences were not obvious for genes involved in other biological activities associated with SM production (Supplementary Figure S7). The BGCs showed enrichment of active histone modifications of H3K9ac and H3K27ac, while being depleted of the repressive modification of H3K27me3 in the putrescine treatment (Supplementary Figure S7). These findings were consistent with the expression patterns observed in the two conditions, indicating a concordance between chromatin accessibility, histone modifications and gene expression within the BGCs.

The overall chromatin structure of *F*. *graminearum* in the two conditions appeared to be very similar (Figure 2B). However, upon examination of the chromatin structure at a higher resolution, we identified several regions that exhibited significant local structural alternations (Figure 2C and Supplementary Figure S8). Notably, among the most prominent regions were those encoding the DON, gramillin and butenolide clusters (Figure 2C). To further investigate the nature of these structural changes in the clusters, we zoomed in on these regions and discovered that all the three clusters were wrapped by one or more ‘V-shape’ domains in the putrescine treatment, with the most prominent case being the butenolide cluster (Figure 2D). This ‘V-shape’ domain resembled a previously defined chromatin jet (hereafter named as jet-like domain) (52) and was not observed in the same regions when the fungus was cultured with NaNO_3_ (Figure 2D). Thus, the jet-like domain, which has not been reported previously in fungi, marks the BGCs with enhanced local chromatin interaction, elevated gene transcription and altered active/repressive epigenetic modifications (Figure 2D and E).

### The jet-like domain is associated with transcription activation and histone acetylation

**Figure 3.**
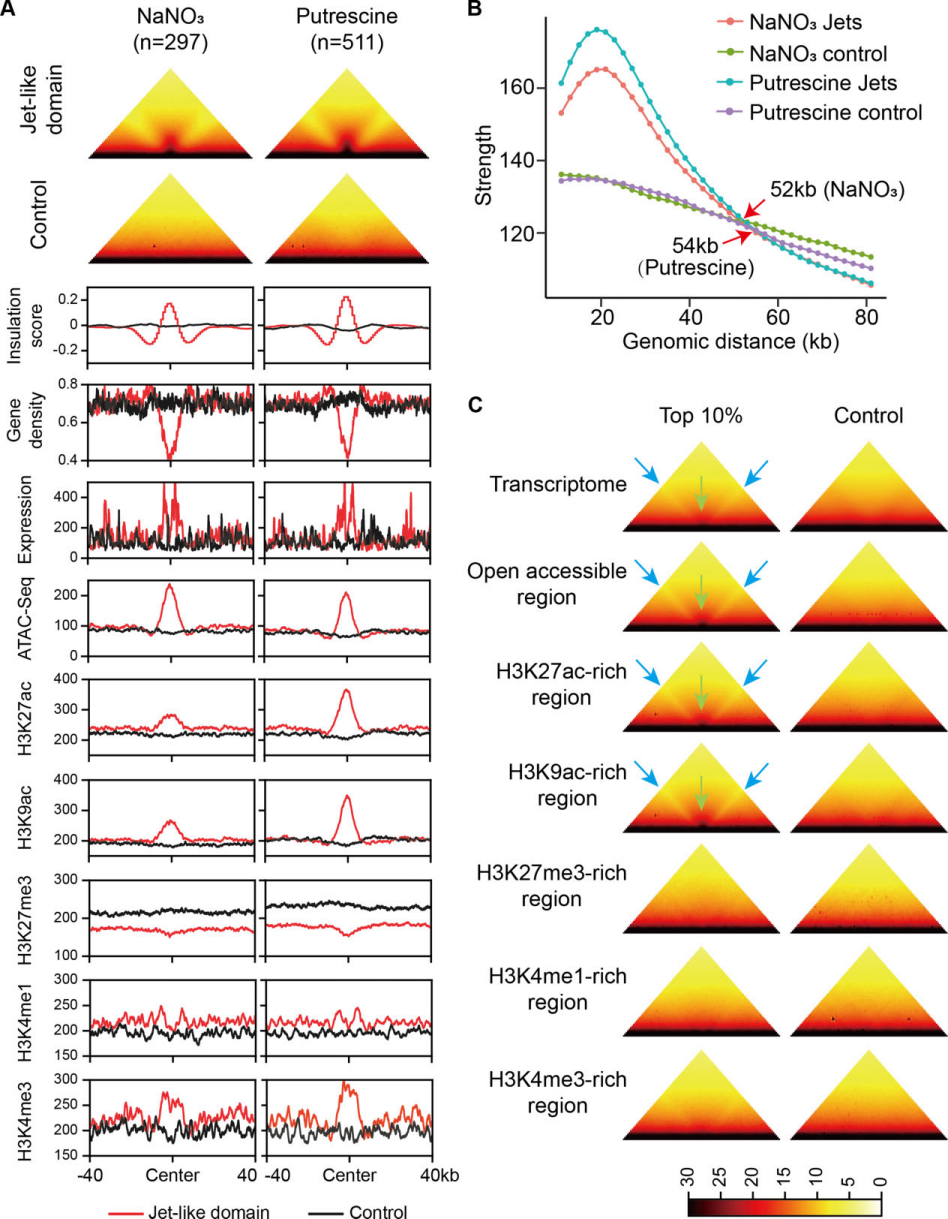
The jet-like domain is associated with active gene transcription and histone acetylation. (**A**) Characterization of the jet-like domains identified under putrescine (*n* = 511) and NaNO_3_ (*n* = 297) conditions. All jet-like domains and their 40-kb upstream and downstream sequences were aggregated to show the Hi-C interactions, insulation score, gene expression, open accessibility and histone modifications. An equal number of randomly selected 80-kb genomic regions lacking jet-like domains were used as a control. (**B**) The strength of Hi-C interactions within a 10 kb × 10 kb square sliding perpendicularly away from the diagonal starting at the center of each jet-like domain or at the center of the randomly selected control regions. Arrows indicate the distance at which interaction strengths are indistinguishable between the jet-like domain and the control. This distance represents the maximum length of the jet-like domain that can propagate in certain culture conditions. (**C**) The top 10% of highly expressed genes or significant peaks of chromosome accessibility and histone modifications were selected, and Hi-C interaction maps (putrescine condition) of the 80-kb window centered at the selected genes or peaks were plotted. Equal numbers of lowly expressed genes or random non-peak regions were selected as the control. A pair of arrows on the sides and a central arrow indicate typical features of the jet-like domain. The resolution for the Hi-C maps (A and C) is 1 kb.

We identified a total of 511 and 297 jet-like domains in the genome of *F*. *graminearum* cultured in putrescine and NaNO_3_ media, respectively (Figure 3A). Chromatin interactions within the jet-like domain displayed a bubble-like structure (Figure 3A), suggesting an enhancement of local physical interactions similar to those observed in the butenolide and other clusters (Figure 2D). The length of bases of the jet-like domains was measured to be ∼8 kb; however, once initiated, the domain could propagate symmetrically for 54 kb in the genome (Figure 3B). Genomic regions encompassed by these domains were characterized by low gene density but exhibited high transcriptional activity. This aligned with the increased chromatin accessibility and the enrichment of histone acetylation marks, H3K9ac and H3K27ac, as well as the promoter-specific H3K4me3 mark (Figure 3A).

To further associate the domain with different genomic features, the top 10% (*n* = 1415) of genes ranked by expression level in the putrescine treatment were selected (Supplementary Table S3), and chromatin interactions within an 80-kb window centered at the translation start site of each gene were plotted. We found that the jet-like domain was observed in genomic regions harboring these highly expressed genes, while other regions containing lowly expressed genes did not show this structure (Figure 3C). Similarly, the jet-like domains were found to be present in the top 10% (*n* = 1529) open accessible regions ranked by fold changes (Supplementary Table S4), while they were absent in the 1529 nonaccessible genomic regions selected at random (Figure 3C). Following the same approach, we found that the jet-like domain was also present in the genomic regions encompassing the top 10% of significant peaks of H3K9ac (*n* = 519; Supplementary Table S5) and H3K27ac (*n* = 485; Supplementary Table S6); however, it showed no or weak correlation with the status of both active and repressive histone methylation marks, H3K4me1 (10%, *n* = 347), H3K4me3 (10%, *n* = 338) and H3K27me3 (10%, *n* = 185) (Figure 3C and Supplementary Tables S7–S9). Importantly, these patterns persisted across both the putrescine and NaNO_3_ treatments (Supplementary Figure S9). In summary, our findings provide compelling evidence supporting the prevalence of the jet-like domain throughout the genome of *F*. *graminearum* and establish its association with elevated level of histone acetylation and enhanced genome open accessibility, both of which are known to facilitate gene transcription.

### Deletion of the HAT gene blocks the formation of the jet-like domain

**Figure 4.**
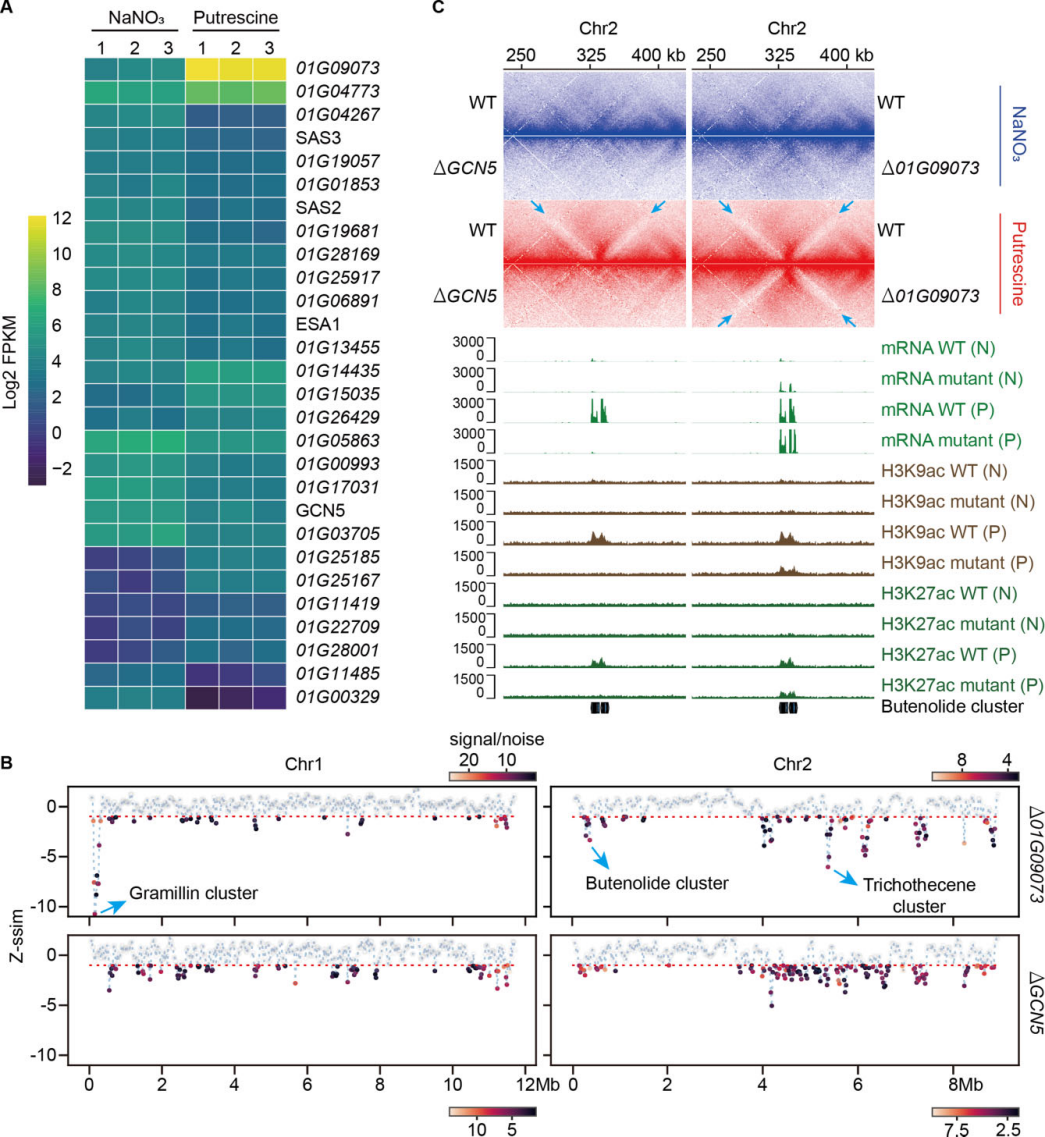
Deletion of *GCN5* blocks the formation of the jet-like domain. (**A**) Genes encoding HATs that were differentially expressed between the two conditions. (**B**) Structure changes of Chr1 and Chr2 in the Δ*01G09073* and Δ*GCN5* mutants under the two culture conditions. Similarity (*z*-normalized similarity score, *Z*-ssim) of Hi-C data generated from the two samples was assessed by CHESS using a sliding window of 100 kb. Highly dissimilar regions (*Z*-ssim ≤ –1) were indicated with the horizontal dash line. (**C**) Hi-C interaction maps of the butenolide cluster in fungi grown in putrescine (P) or NaNO_3_ (N) medium. Arrows indicate the jet-like domain encompassing the BGC. The status of gene expression and histone modifications was calculated from all biological replicates. The resolution for the Hi-C maps is 1 kb.

Because histone acetylation influences chromatin open accessibility, we hypothesized that it plays a critical role in the formation of the jet-like domain. We focused on the HATs, which are enzymes responsible for histone acetylation. Through domain search and phylogenetic analysis, we identified 73 putative HATs in the PH-1 genome (Supplementary Figure S10 and Supplementary Table S10), among which 28 genes showed differential expression between the two conditions (Figure 4A). Consequently, our study focused on two specific HATs, namely GCN5 and 01G09073. GCN5 is a central enzyme for histone acetylation and interacts with a variety of regulatory proteins to control gene expression and nutrient adaptation (20,69), whereas the gene *01G09073*, located within the butenolide cluster and encoding a functionally uncharacterized homolog of yeast HPA2/3 (Supplementary Figure S10), was drastically upregulated by 169-fold in the putrescine treatment (Figure 4A). We created gene deletion mutants for both HATs, and performed RNA-seq, Hi-C sequencing and CUT&Tag sequencing of H3K9ac and H3K27ac under both putrescine and NaNO_3_ conditions (Figure 4B and C, Supplementary Figure S11 and Supplementary Table S2). Genomic regions encoding the gramillin, butenolide and trichothecene clusters were the most significantly reorganized in the wild-type (WT) strain in the putrescine treatment (Figure 2C). These regions also showed pronounced local reorganization in the Δ*01G09073* mutant under the two conditions, although the extent of reorganization for butenolide and trichothecene clusters seemed to be slightly attenuated compared to the WT strain (Figure 4B). In contrast, none of the local reorganizations in the three clusters had occurred in the Δ*GCN5* mutant, suggesting that *GCN5* mediated local chromosome reorganization of *F*. *graminearum* in response to putrescine.

Next, we revisited the genomic regions harboring the butenolide cluster, where the presence of the jet-like domain was initially observed in the WT strain under the putrescine condition (Figure 2D). We found that the jet-like domain was absent in the WT and both mutants under the NaNO_3_ culture condition; however, it became very clear when the WT and Δ*01G09073* mutant were cultured in the putrescine treatment (Figure 4C). In contrast, the jet-like domain was completely absent in the Δ*GCN5* mutant under the putrescine condition, corroborating previous observations regarding chromosomal reorganization patterns (Figure 4B). Furthermore, gene transcription and the deposition of H3K9ac and H3K27ac within the butenolide cluster remained inducible in the Δ*01G09073* mutant in response to putrescine, whereas such inducibility was entirely abolished in the Δ*GCN5* mutant (Figure 4C). Similar patterns were also observed for the gramillin and trichothecene clusters (Supplementary Figure S11). Overall, our data substantiated the involvement of *GCN5* in the establishment of the jet-like domain in *F*. *graminearum*.

### The jet-like domain enhances transcription of exogenous gene

The presence of the jet-like domain has sparked inquiries into its potential utility for enhancing expression of exogenous genes in future genome engineering of this fungus. The Δ*01G09073* mutant strain was considered a good system to study this, as this gene was housed within the butenolide cluster, and its replacement with hygromycin resistance gene cassette (*hph*) in the Δ*01G09073* mutant did not block the formation of the jet-like domain surrounding the cluster (Figure 4C). We calculated the expression of *hph* in the Δ*01G09073* mutant under the two culture conditions, representing the presence or absence of the jet-like domain surrounding itself. We found that the expression of *hph* was increased by 2.5-fold (*P*= 1.5E−17) in the presence of the jet-like domain (Supplementary Table S11). Moreover, the growth inhibition rate for the Δ*01G09073* mutant treated with 100 μg/ml hygromycin B was decreased by 27.7% (*P*< 0.01) when cultured in the putrescine medium with the jet-like domain (Supplementary Figure S12). These data suggest that forced chromatin looping as exampled by the formation of the jet-like structure can reprogram the expression of exogenous genes in *F*. *graminearum*.

### The HAT 01G09073 is a new regulator of DON biosynthesis

**Figure 5.**
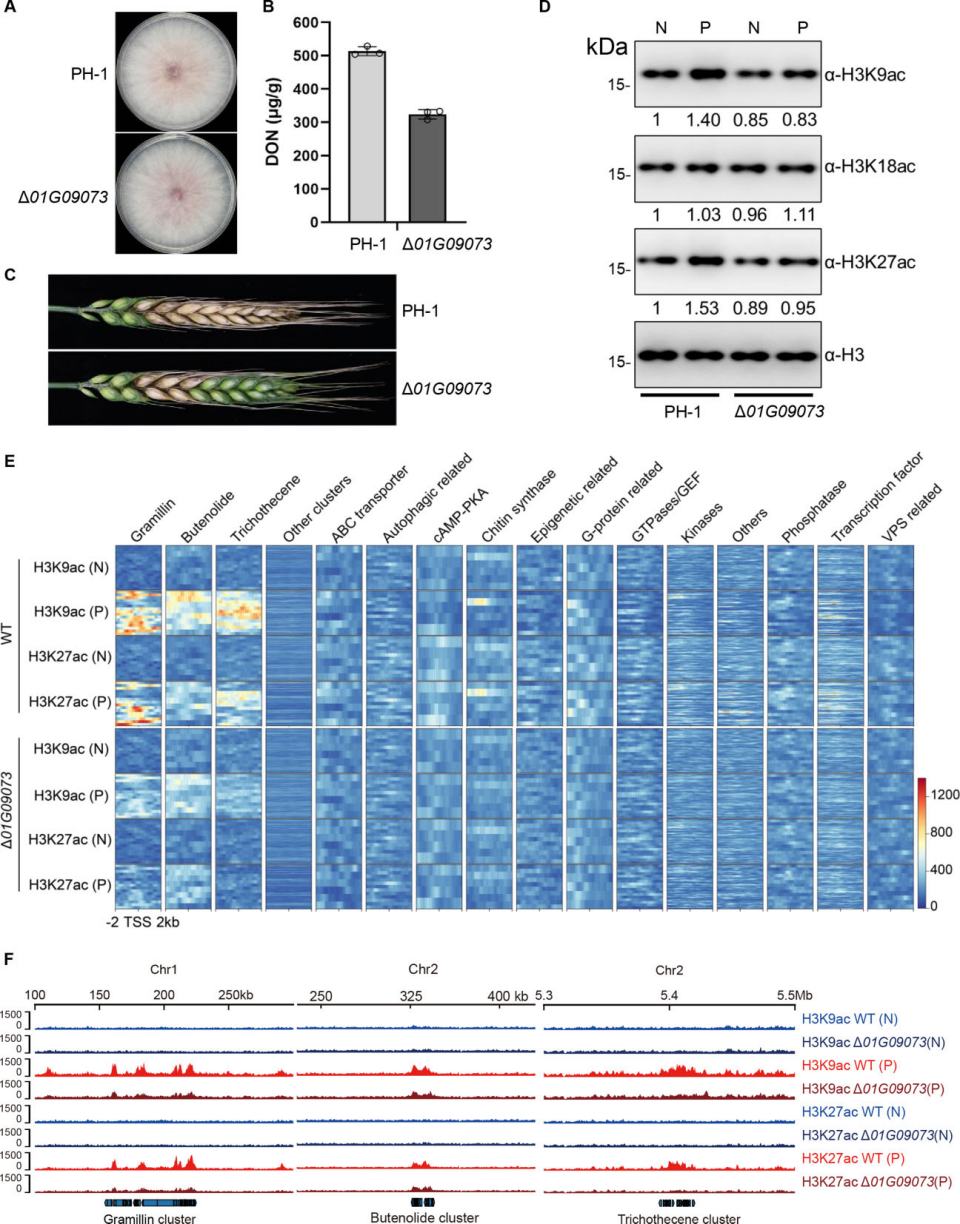
The HAT 01G09073 is a new regulator of DON biosynthesis. (**A**) Colonies of the Δ*01G09073* and WT strains grown on the PDA medium for 3 days. (**B**) DON production in the mutant and WT strains after cultivation in the putrescine medium for 5 days. (**C**) Virulence assay of the two strains. Photos were taken 15 days after inoculation. (**D**) Western blots showing the levels of H3K9ac, H3K18ac and H3K27ac in the mutant and WT strains. Values below each gel figure are the intensities of detected protein bands relative to that of H3 band after immunoprecipitation. (**E**) Levels of H3K9ac and H3K27ac on genes relevant to different functional categories. (**F**) Levels of H3K9ac and H3K27ac on genomic regions housing gramillin, butenolide and trichothecene clusters in the Δ*01G09073* mutant and the WT strain under the putrescine (P) or NaNO_3_ (N) condition. Biological replicates were combined to calculate the values.

Due to the pronounced upregulation of *01G09073* in the DON-inducing medium, we embarked on further investigations to elucidate its underlying biological functions. The Δ*01G09073* mutant showed no obvious growth defect compared to the WT (Figure 5A). However, DON production in the Δ*01G09073* mutant was decreased by ∼37% (Figure 5B). Consistently, the mutant showed attenuated virulence on wheat in comparison to the WT (Figure 5C). Phylogenetic analysis indicated the close relationship between *01G09073* encoding acetyltransferase and GCN5 (Supplementary Figure S10). GCN5 acts as the catalytic subunit responsible for acetylating various lysine residues on different histone subunits, including H2BK11, H2BK16, H3K9, H3K14, H3K18, H3K23 and H3K27 (70,71). We examined the acetylation status of H3K9, H3K18 and H3K27 in the Δ*01G09073* mutant through western blotting. Our results showed that the levels of H3K9ac and H3K27ac in the mutant slightly decreased compared to the WT under the NaNO_3_ treatment. In the putrescine treatment, a significant enhancement of H3K9ac and H3K27ac was observed in the WT, whereas the *Δ01G09073* mutant failed to show such increase (Figure 5D). In contrast, the level of H3K18ac remained largely unaltered in both strains and under all conditions examined.

We categorized genes based on their putative functions to explore the potential link between gene functions and the induced histone acetylation (H3K9ac and H3K27ac) mediated by *01G09073*. Interestingly, we found that deletion of *01G09073* resulted in a significant reduction in the acetylation levels of H3K9 and H3K27 within the biosynthetic clusters of gramillin, trichothecene and butenolide, as well as their associated regulatory factors (Figure 5E and F). It is worth noting that the substrate specificity and precise mechanism of action for *01G09073* are yet to be fully elucidated. Nevertheless, our findings strongly indicate that *01G09073* exerts regulatory control over the BGCs of *F*. *graminearum* through its modulation of histone acetylation, such as H3K9ac and H3K27ac. However, such regulatory effects are dependent on specific environmental conditions.

## Discussion

Regulation of BGCs involves intricate control at multiple levels, including transcriptional and post-transcriptional regulation (19,20). In this study, we demonstrate the potential regulation of 3D chromosomal architecture and BGC activity, particularly in the context of the BGC responsible for the DON toxin production (Figure 2). Active BGCs exhibit a jet-like domain (Figure 2), and this spatial arrangement likely facilitates gene transcription by enabling close proximity to transcriptional resources within the nuclear milieu. Importantly, our findings establish a direct association between the presence of the jet-like domain and histone acetylation marks (Figure 3), and reveal that knockout of *GCN5* disrupts the formation of the jet-like domain (Figure 4). Intriguingly, we find that incorporation of an exogenous gene within the jet-like domain leads to a significant upregulation of its expression (Supplementary Figure S12). These findings provide evidence for an additional regulatory mechanism underlying the biosynthesis and regulation of secondary metabolism in fungi. These results also underscore the potential utility of leveraging the 3D organization of chromosomes to enhance the efficiency of genome engineering in fungal species.

Euchromatin and heterochromatin typically segregate into distinct A/B nuclear compartments in metazoans. This spatial organization arises from the fact that genomic regions with similar transcriptional activities tend to engage in spatial interactions within the nucleus (9,72). Investigation into A/B compartments in fungi has unveiled intriguing insights into genome compartmentalization. For instance, the arbuscular mycorrhizal fungus, *Rhizophagus irregularis*, manifests clearly defined A/B compartments that align with transcriptional activity and repeat content (73). Conversely, other fungi show minimal chromatin compartmentalization (74). In our study, we find that genome compartmentalization of *F*. *graminearum* reflects a complex interplay of heterochromatic regions and ancient genome rearrangements (Figure 1A). Notably, aggregation of heterochromatic regions being apart from euchromatin has been observed in numerous fungi (12,75–77).

The fungal genomes also have TAD-like structures (12,13,76,78,79), which span several hundreds of kilobases, and their boundaries in yeast are associated with gene regulation and DNA replication (78). Furthermore, the chromatin loops or its analogous form globules are absent in some fungi (74,47) but present in the fission yeast (12,80) and *Neurospora crassa* (76). In our examination of *F*. *graminearum*, we identified TAD-like domains; however, their strength was notably weaker in comparison to metazoans. Typic chromatin loops were not observed in *F*. *graminearum*. However, we discovered a jet-like domain that is prevalent in the genome of *F*. *graminearum*. Chromatin jets and analogous structures, such as plumes, fountains and ‘hinge-like’ domains, have been observed in mammals (52,81), worms (82,83) and zebrafish (84,85). There is evidence suggesting that these structures arise from specific cohesin loading in narrow accessible chromatin regions (52). While CTCF (CCCTC-binding factor), a blocker of cohesin, is not necessary for jet formation, it can impact cohesin looping and thus shape the domain structure (52,83). Consistently, genomes with or without CTCF proteins exhibit jet-like chromatin structures. The precise mechanisms driving preferential cohesin loading remain unclear. Emerging studies indicate that this targeted loading occurs at enhancers, and the absence of certain pioneer transcription factors, which establish open chromatin regions and facilitate H3K27 acetylation on enhancers, can influence the formation of the jet-like domain (83). Moreover, H3K27ac-associated BET proteins interact with cohesin motors, and thus can affect the loading of cohesin (86,87). In an effort to determine whether cohesin is also involved in the formation of the jet-like domain in *F*. *graminearum*, we attempted to create knockout mutants for the core cohesin subunits, including SMC1, SMC3, SCC1, SCC3, SCC2 and SCC4. However, despite examining dozens to hundreds of transformants, we were unable to obtain true mutants for these genes, implying their essential nature for this fungus. Thus, the mechanisms behind the formation of the jet-like domain in *F*. *graminearum* remain to be explored.

Enhanced histone acetylation at BGC loci is frequently correlated with active BGCs, leading to increased expression of genes within the cluster. In many fungal species, including *F. graminearum*, GCN5 has been implicated in the activation of BGCs through its regulatory influence on chromatin accessibility (20). However, the precise mechanisms underlying the GCN5-mediated changes in chromatin structure remain poorly characterized. Our data uncovered a pivotal role of GCN5 in the establishment of the jet-like domain in *F. graminearum*. This newly identified jet-like domain constitutes a paradigmatic framework for comprehending the spatial regulation of BGCs orchestrated by GCN5. In addition, we identified a new HAT that is involved in the regulation of DON biosynthesis. This gene is located within the butenolide cluster and strongly induced under the DON-stimulating conditions. Since the BGCs of butenolide and trichothecene are often co-regulated, this gene might have been co-opted to ensure the proper regulation of DON production in response to internal and external signals.

In conclusion, we provide comprehensive insights into the 3D chromosomal organization, epigenetic modifications, chromatin accessibility and gene expression dynamics in *F*. *graminearum* during DON production. The observations of the jet-like domains, their association with specific genomic features and the role of HATs highlight the complex interplay between chromatin structure, epigenetic modifications and gene regulation. These findings contribute to our understanding of the molecular mechanisms underlying fungal secondary metabolism and open up potential avenues for future genome engineering and gene expression manipulation in eukaryotes.

## Supplementary Material

gkae131_Supplemental_Files

## Data Availability

Raw reads generated in this study have been deposited in the National Center for Biotechnology Information BioProject database under accession no. PRJNA995191.
