# Supplementary material for: The jet-like chromatin structure defines active secondary metabolism in fungi
Source: Nucleic Acids Res. 2024 Feb 26;52(9):4906–21. doi: 10.1093/nar/gkae131 (PMC11109943; doi:10.1093/nar/gkae131)
Supplement: gkae131_Supplemental_Files [file gkae131_supplemental_files.zip › Supplementary Table Legends.pdf]

Supplementary Table Legends

Table S1. Primers used in this study.

Table S2. Statistics of libraries generated in this study.

Table S3. Differentially expressed genes in *F. graminearum* PH-1 under different treatments. Expression of each gene is represented by the FPKM value.

Table S4. Open chromatin regions (OCRs) in *F. graminearum* genome under different treatments. OCRs were identified based on the ATAC-Seq data.

Table S5. Peaks of H3K9ac modification in *F. graminearum* genome under different treatments.

Table S6. Peaks of H3K27ac modification in *F. graminearum* genome under different treatments.

Table S7. Peaks of H3K4me1 modification in *F. graminearum* genome under different treatments.

Table S8. Peaks of H3K4me3 modification in *F. graminearum* genome under different treatments.

Table S9. Peaks of H3K27me3 modification in *F. graminearum* genome under different treatments.

Table S10. Expression and functional annotation of histone acetyltransferases in *F. graminearum* genome. Expression of each gene is represented by the FPKM value.

Table S11. Differentially expressed genes in the *Δ01G09073* and *ΔGCN5* mutants under the two treatments.
