# Supplementary material for: The jet-like chromatin structure defines active secondary metabolism in fungi
Source: Nucleic Acids Res. 2024 Feb 26;52(9):4906–21. doi: 10.1093/nar/gkae131 (PMC11109943; doi:10.1093/nar/gkae131)
Supplement: gkae131_Supplemental_Files [file gkae131_supplemental_files.zip › Supplementary_Figures_20240203.pdf]

# Supplemental Information

## The jet-like chromatin structure defines active secondary metabolism in fungi

Wenyong Shao[1,†], Jingrui Wang[1,†], Yueqi Zhang[1], Chaofan Zhang[1,2], Jie Chen[3], Yun Chen[1], Zhangjun Fei[4], Zhonghua Ma[1,*], Xuepeng Sun[2,*], Chen Jiao[1,*]

[1]State Key Laboratory of Rice Biology, Key Laboratory of Molecular Biology of Crop Pathogens and Insects, Institute of Biotechnology, Zhejiang University, Hangzhou 310058, Zhejiang, China. [2]Collaborative Innovation Center for Efficient and Green Production of Agriculture in Mountainous Areas of Zhejiang Province, College of Horticulture Science, Zhejiang A&F University, Hangzhou 311300, Zhejiang, China. [3]National Joint Engineering Laboratory of Biopesticide Preparation, College of Forestry and Biotechnology, Zhejiang A&F University, Hangzhou 311300, Zhejiang, China. [4]Boyce Thompson Institute, Cornell University, Ithaca 14853, NY, USA.

*To whom correspondence should be addressed. Email: biochenjiao@zju.edu.cn
Correspondence may also be addressed to Xuepeng Sun. Email: xs57@zafu.edu.cn
Correspondence may also be addressed to Zhonghua Ma. Email: zhma@zju.edu.cn

[†]The authors wish it to be known that, in their opinion, the first two authors should be regarded as Joint First Authors.

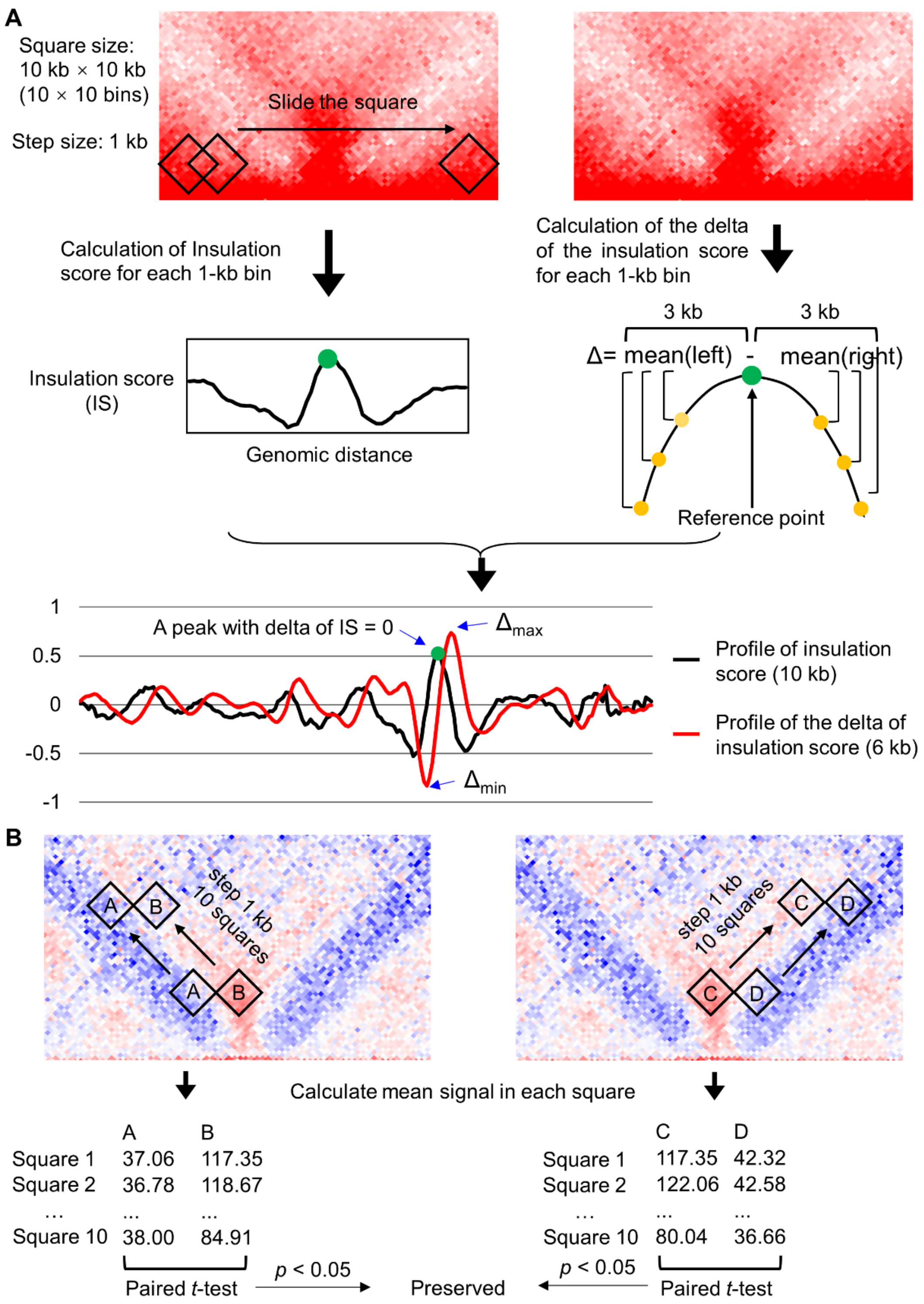


**Figure S1.** Illustration of the methods used for identifying jet-like domains. (**A**) The insulation score for each 1-kb bin was calculated by sliding a 10 kb × 10 kb square along the matrix diagonal. The normalized mean signal within the square was then assigned to

each bin (left panel). The right panel depicts calculation of the delta value, which is defined as the difference between the mean insulation scores 3 kb to the left of the central bin and 3 kb to the right of the central bin. Bins at the peak of insulation score profile with a delta value equal to zero were extracted. For these bins, the strength of insulation was further calculated by measuring the difference of delta values between the local maximum ($\Delta_{max}$) and local minimum ($\Delta_{min}$) of the bin (lower panel). (**B**) Illustration of the methods for verification of the stripes surrounding the domain.

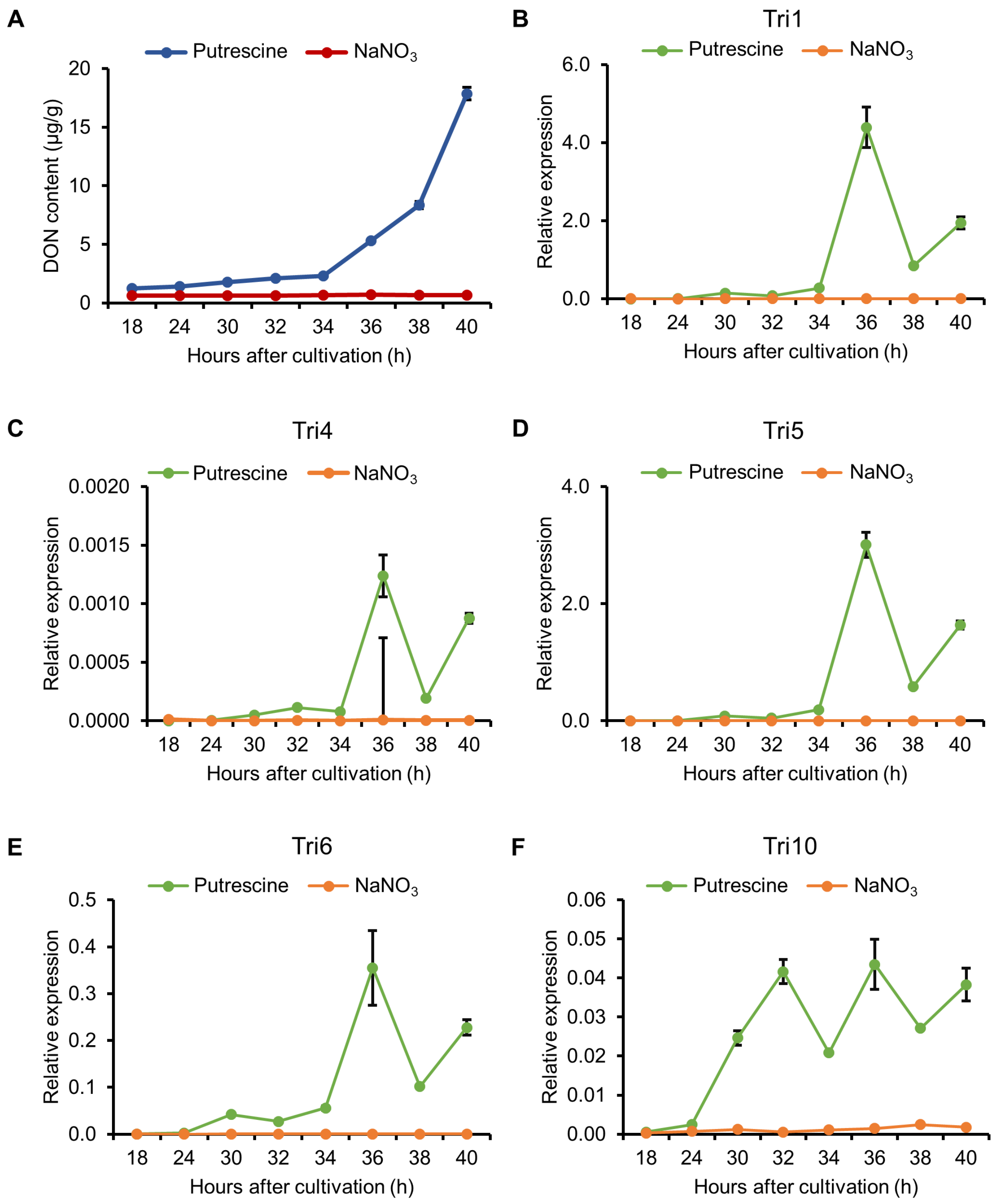


**Figure S2.** Measurement of DON production and gene expression of trichothecene cluster. Mycelia of PH-1 were collected at 8 timepoints to quantify their DON production **(A)** as well as gene expression of key components of the trichothecene cluster **(B-F).**

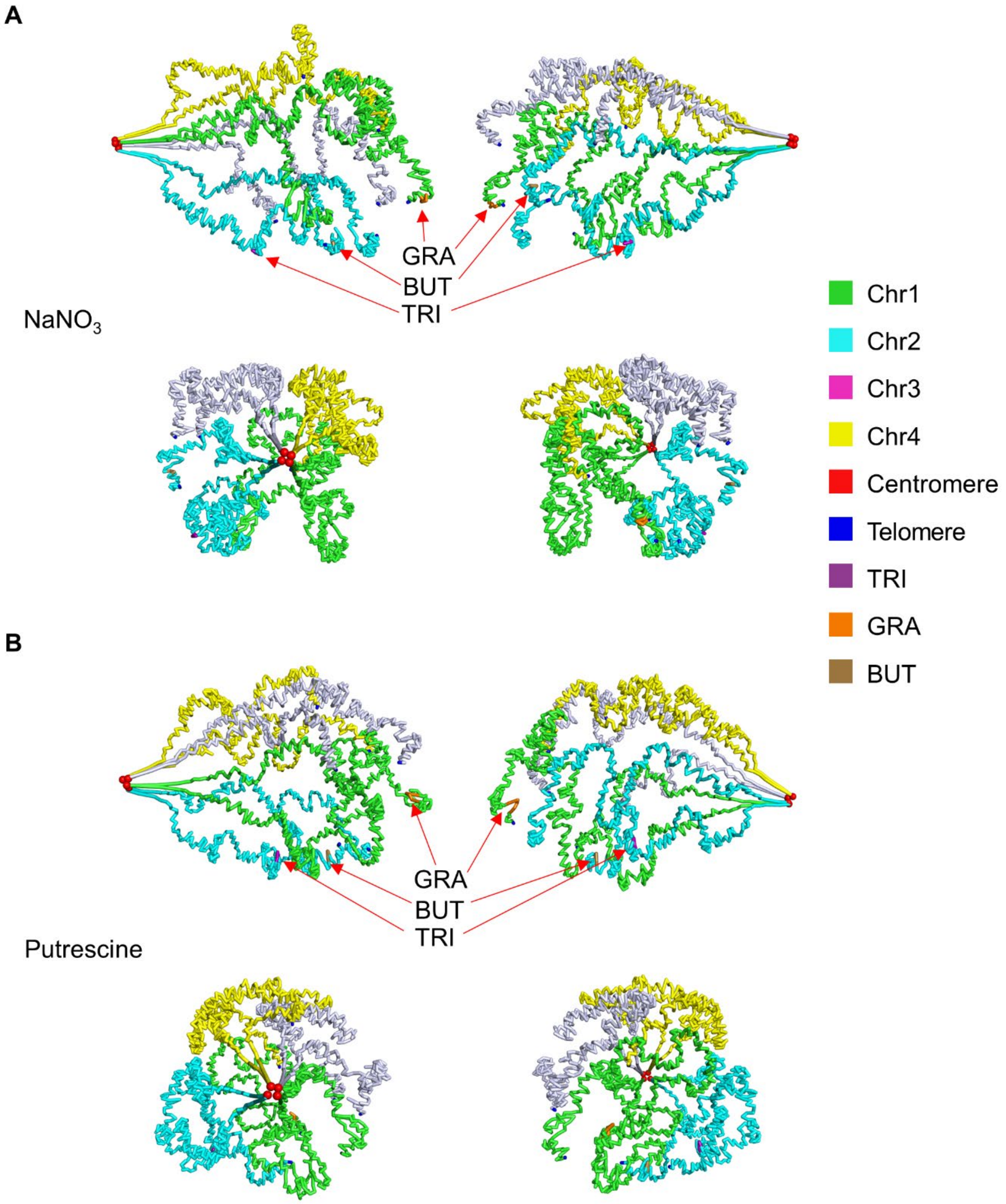


**Figure S3.** Modelled 3D structure of *F. graminearum* genome cultured under the two conditions. The contact matrix was computed with a resolution of 1-kb, and every 20 bins were combined to create the matrix used for modeling. TRI, trichothecene cluster; GRA, gramillin cluster; BUT, butenolide cluster.

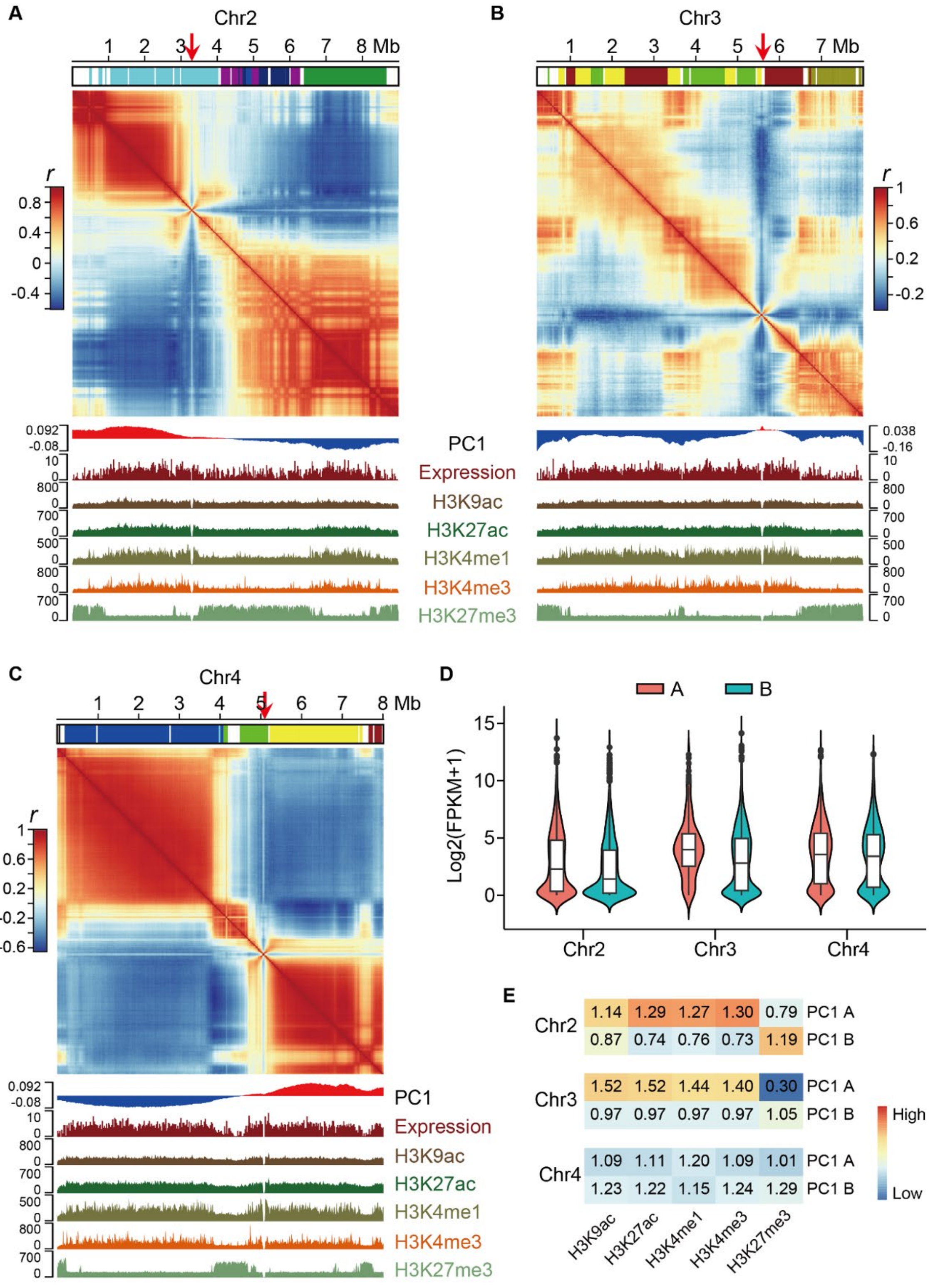


**Figure S4.** Epigenetic modifications and three-dimensional structure of *F. graminearum* genome. **(A-C)** Epigenetic modifications and compartmentalization of the three chromosomes. The heatmap shows Pearson correlation coefficient (*r*) of Hi-C interactions

on chromosomes at the 25-kb resolution. Colored bar on the top represents the genome synteny between *F. graminearum* and *F. oxysporum*, and each color indicates a single chromosome of *F. oxysporum* shown in **Supplementary Figure S5**, while the white color indicates no synteny. Red arrow points to the centromere position. PCA-based compartmentalization as well as the status of gene expression and epigenetic modifications of each 25-kb window are displayed on the bottom. Biological replicates are combined to generate the plot. **(D)** Distribution of gene expression in A/B compartments defined by the first principal component (PC1). Expression of each gene was calculated as the averaged log2(FPKM+1) of three biological replicates. **(E)** Enrichment analysis of epigenetic modifications in A/B compartments. Values indicate the fold of overrepresentation or underrepresentation of each marker in corresponding compartment.

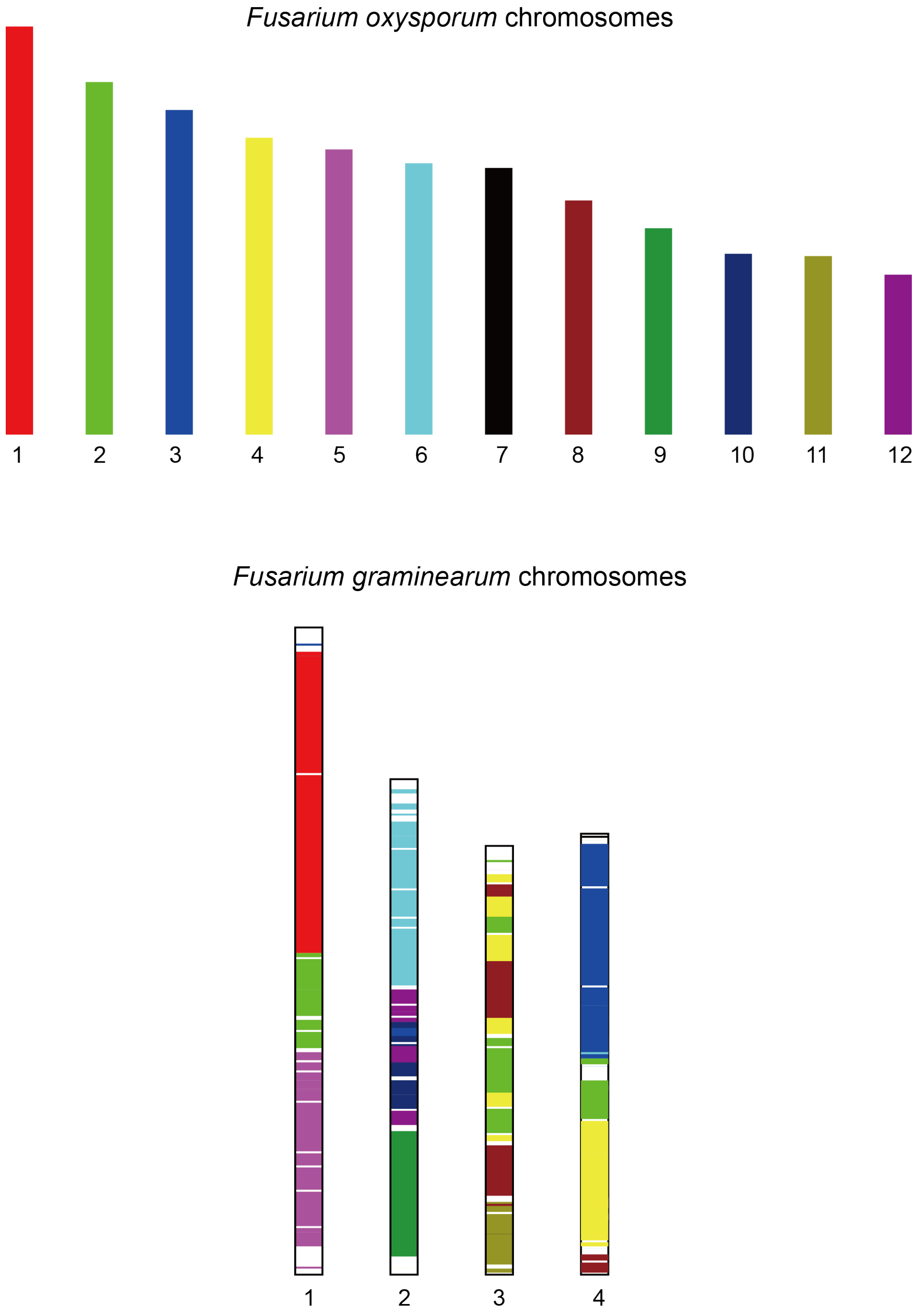


**Figure S5.** Barplot showing the genome synteny between *F. graminearun* and *F. oxysporum*. Each chromosome of *F. oxysporum* was labeled with a different color, and chromosomes of *F. graminearun* were colored based on their synteny with *F. oxysporum* genome. White color indicates no synteny.

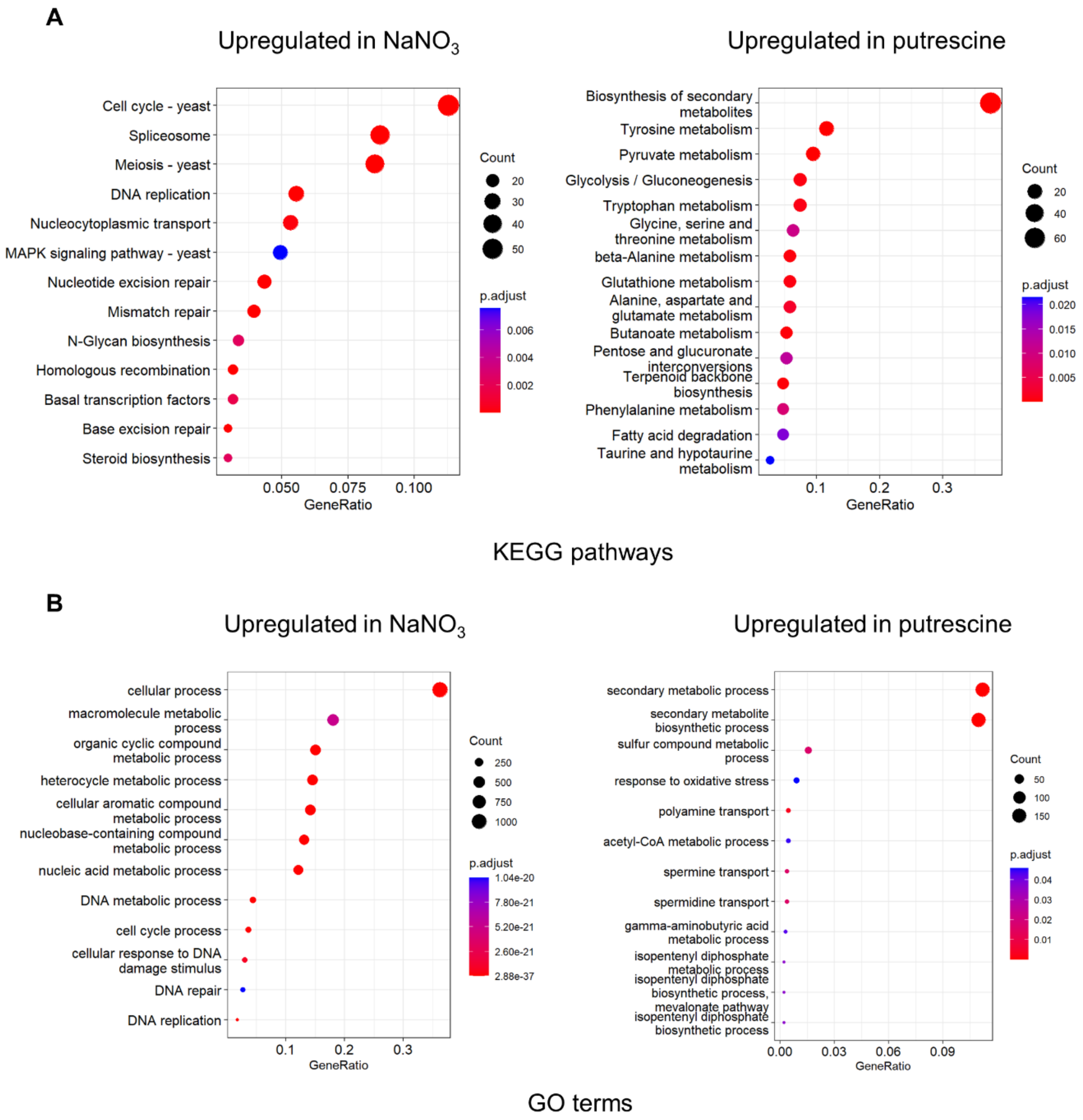


**Figure S6.** Enrichment analysis of KEGG pathways (**A**) and GO terms (**B**) in the differentially expressed genes of PH-1 cultured in the two media.

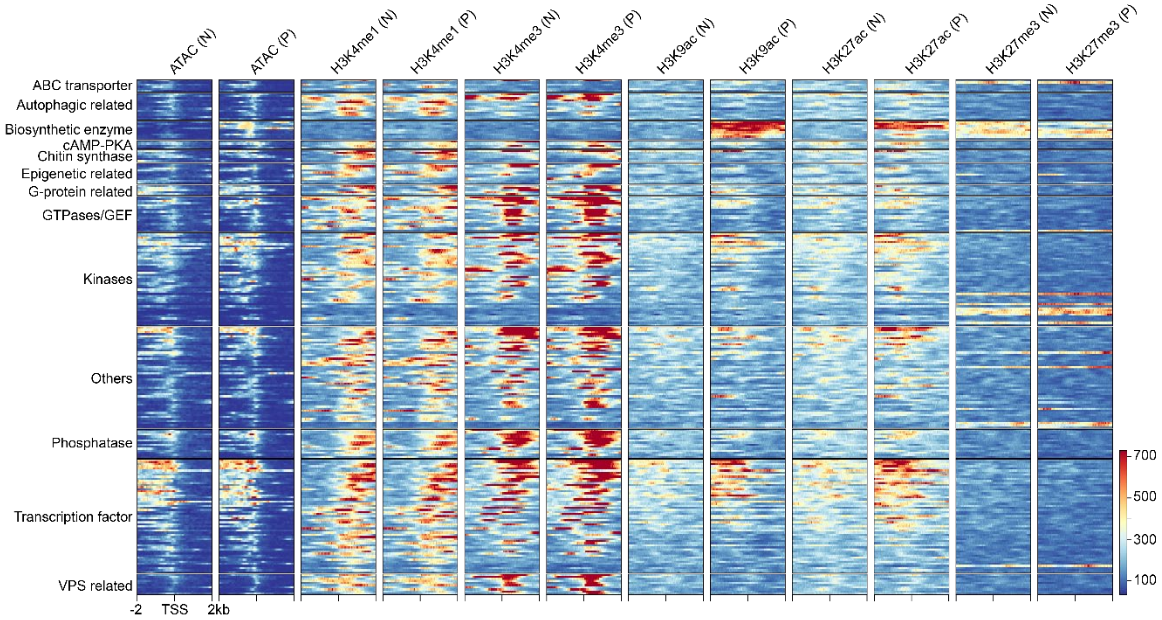


**Figure S7.** Status of chromatin accessibility and histone modifications on genes relevant to each functional category.

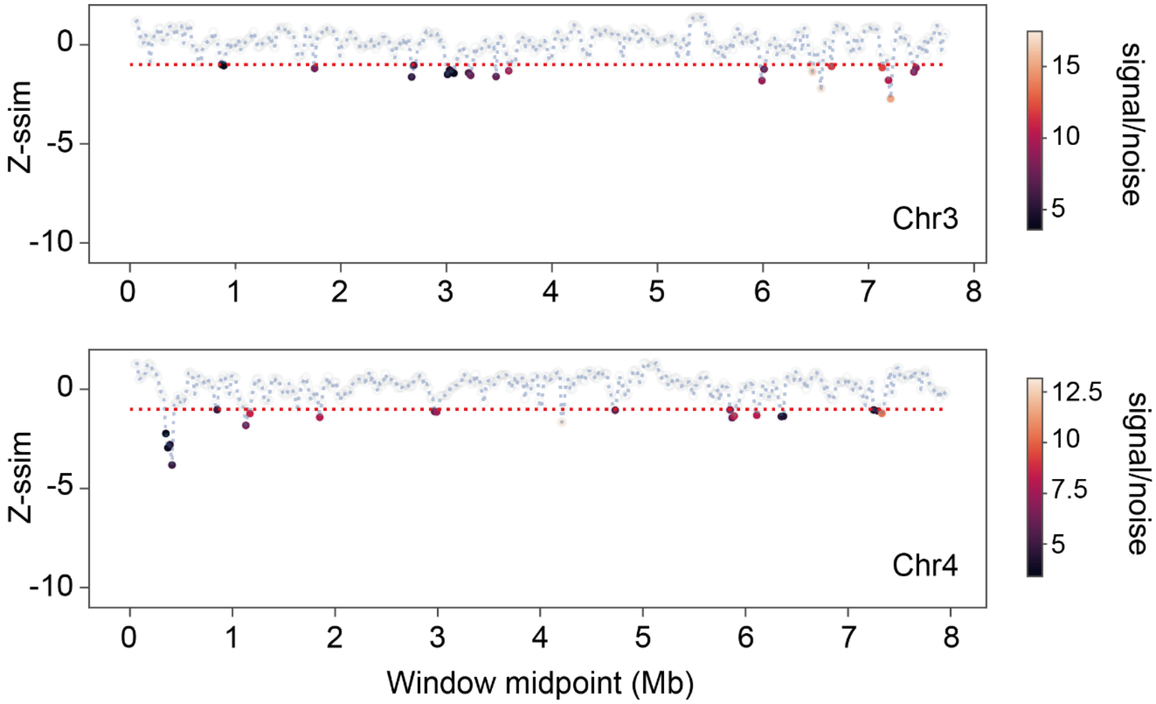


**Figure S8.** Structure changes of Chr3 and Chr4 under the two culture conditions. Similarity (z-normalized similarity score, Z-ssim) of Hi-C data generated from the two samples were assessed by CHESS based on a window of 100-kb. Highly dissimilar regions (Z-ssim ≤ -1.2) were indicated with the red dash line.

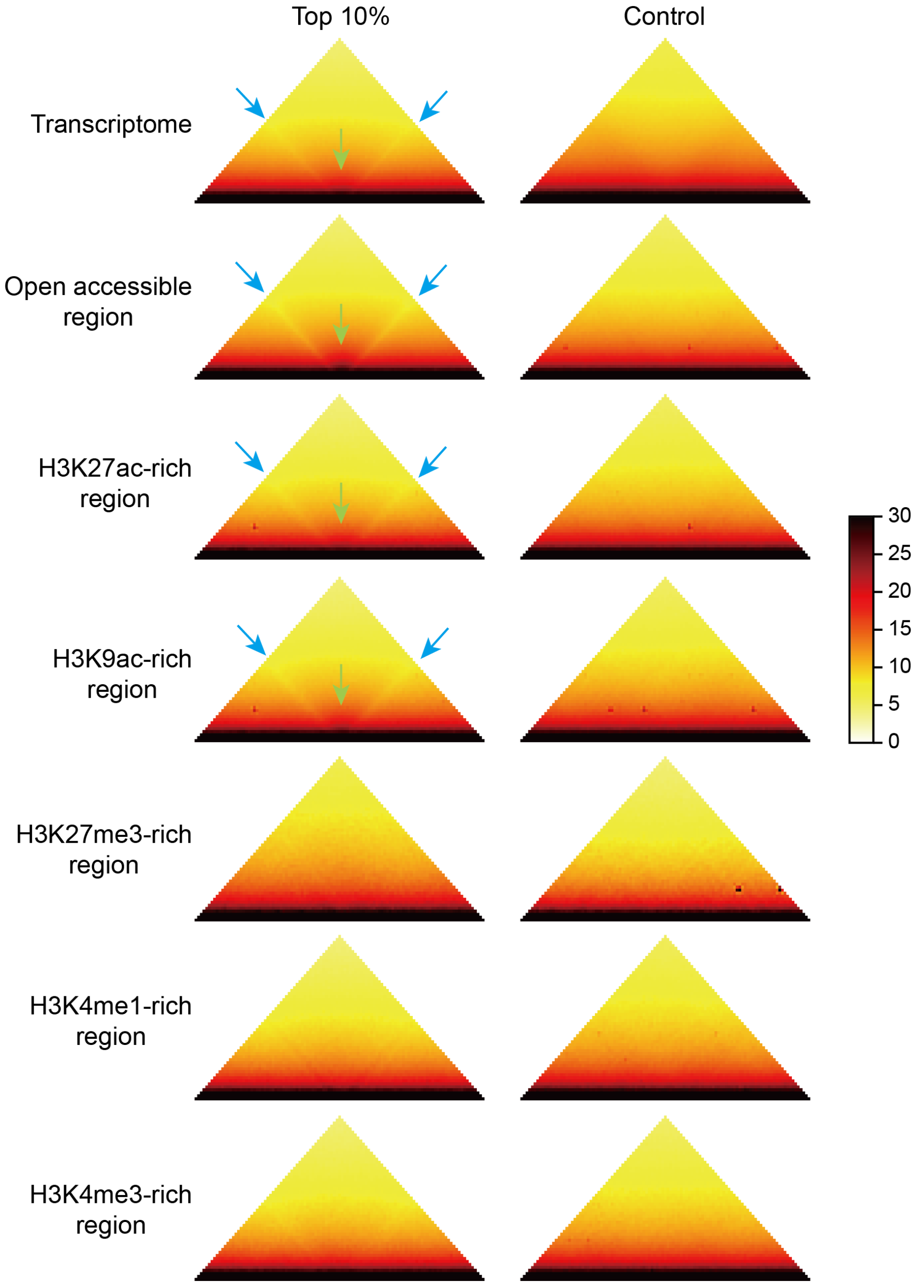


**Figure S9.** The jet-like domain is associated with active gene transcription and histone acetylation. The top 10% highly expressed genes or most significant peaks of chromosome accessibility and histone modifications were selected, and Hi-C interaction maps ($NaNO_3$ condition) of the 80-kb window centered at the selected genes or peaks were plotted. Equal numbers of lowly expressed genes or random non-peak regions were selected as the control. Blue and green arrows indicate the typical structure of the jet-like domain. The resolution for the Hi-C map is 1 kb.

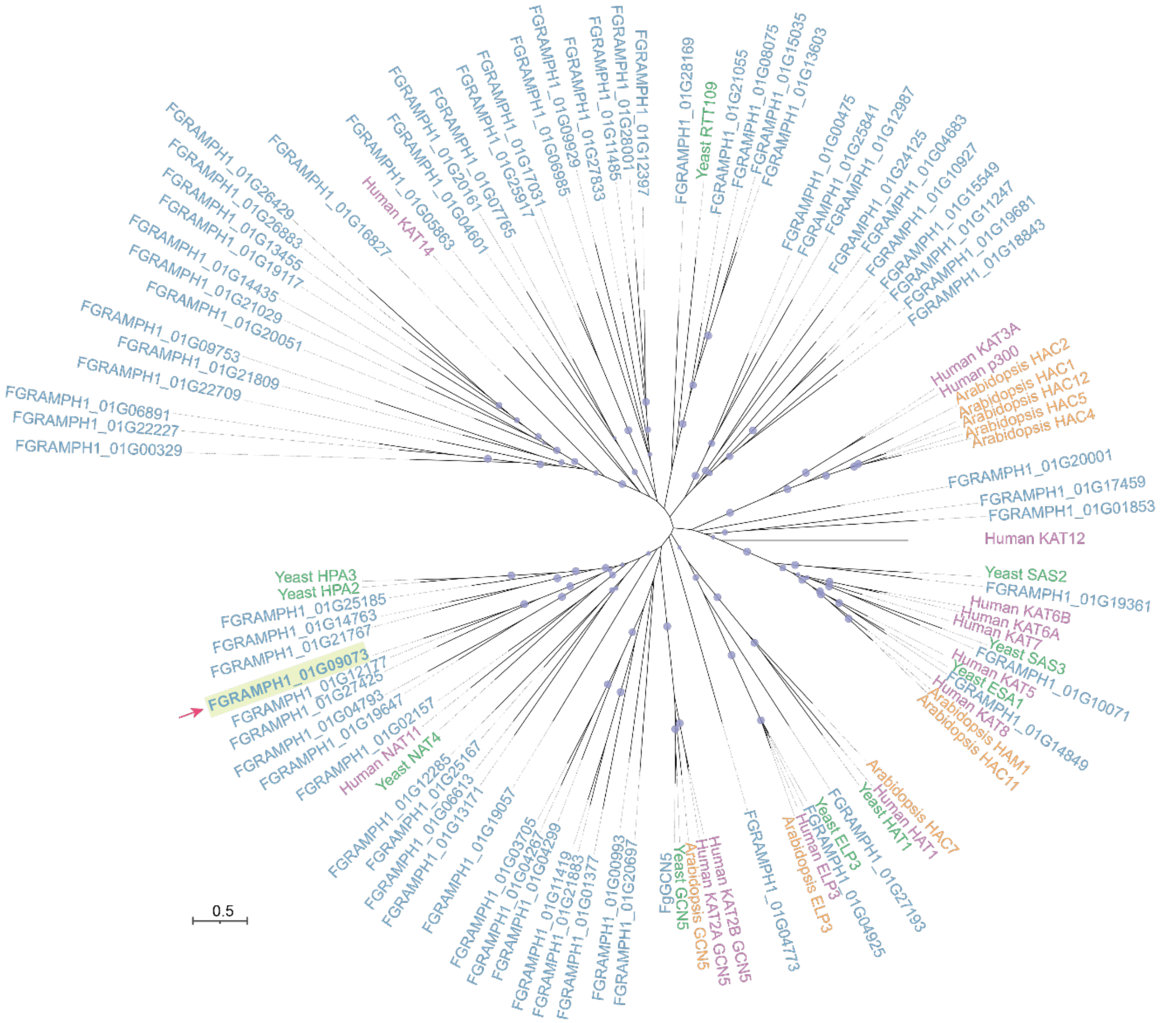


**Figure S10.** Unrooted phylogenetic tree of predicted histone acetyltransferase from *F. graminearun* and known counterparts from human, Arabidopsis, and yeast. Genes from *F. graminearun* were in blue color. The maximum likelihood phylogeny was constructed using ‘VT+I+G4’ model with 1000 bootstrap replicates. Pies on branches indicate bootstrap support greater than 80%.

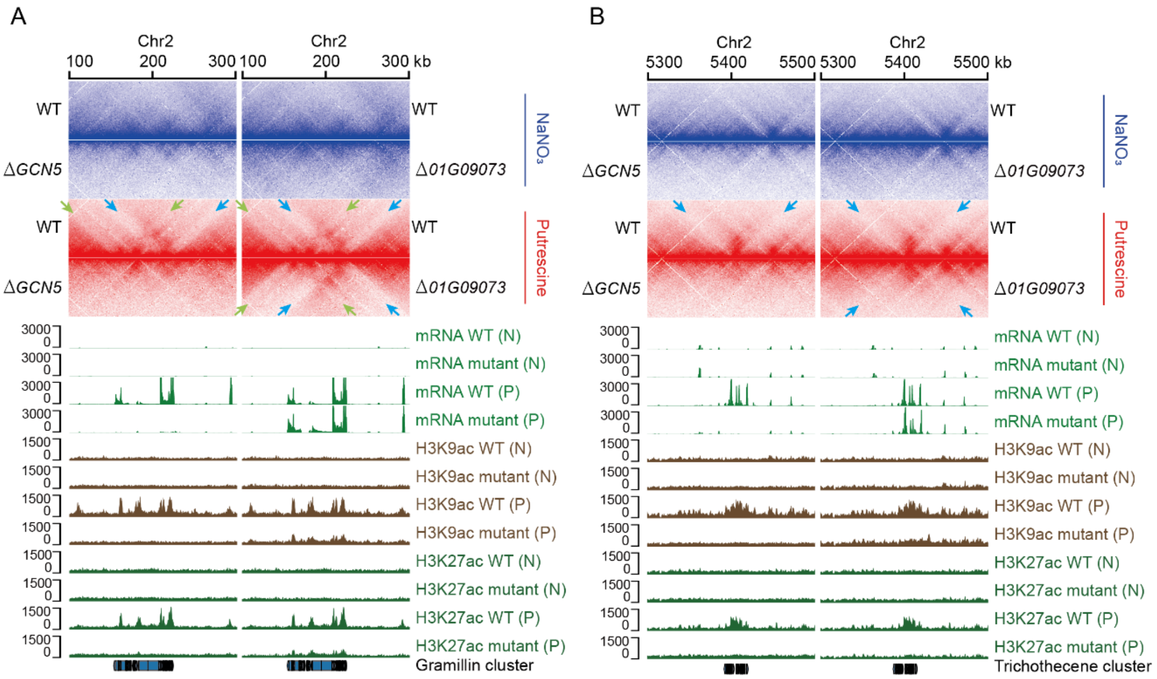


**Figure S11.** Hi-C interaction maps of the gramillin (**A**) and trichothecene (**B**) clusters in fungi grown in putrescine (P) or $NaNO_3$ (N) medium. Arrows indicate the jet-like domain encompassing the BGCs. The status of gene expression and histone modifications was calculated from all biological replicates. The resolution for the Hi-C maps is 1 kb.

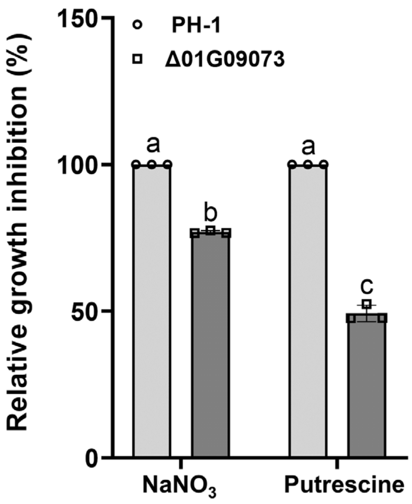


**Figure S12.** Relative growth inhibition of the mutant and WT strains under different culture media.
